# Synergistic effect of potential alpha-amylase inhibitors from Egyptian propolis with acarbose using in silico and in vitro combination analysis

**DOI:** 10.1186/s12906-024-04348-x

**Published:** 2024-01-30

**Authors:** Ahmed A. Nada, Aly M. Metwally, Aya M. Asaad, Ismail Celik, Reham S. Ibrahim, Safa M. Shams Eldin

**Affiliations:** 1 Department of Pharmacognosy, Faculty of Pharmacy, Alexandria University, Alkhartoom Square, Alexandria, 21521 Egypt; 2 Department of Pharmaceutical Chemistry, Faculty of Pharmacy, Erciyes University, Kayseri, 38039 Turkey

**Keywords:** Alpha-amylase, Combination analysis, Cooperative binding, Egyptian propolis, GC–MS

## Abstract

**Background:**

Type 2 Diabetes mellitus (DM) is an affliction impacting the quality of life of millions of people worldwide. An approach used in the management of Type 2 DM involves the use of the carbohydrate-hydrolyzing enzyme inhibitor, acarbose. Although acarbose has long been the go-to drug in this key approach, it has become apparent that its side effects negatively impact patient adherence and subsequently, therapeutic outcomes. Similar to acarbose in its mechanism of action, bee propolis, a unique natural adhesive biomass consisting of biologically active metabolites, has been found to have antidiabetic potential through its inhibition of α-amylase. To minimize the need for ultimately novel agents while simultaneously aiming to decrease the side effects of acarbose and enhance its efficacy, combination drug therapy has become a promising pharmacotherapeutic strategy and a focal point of this study.

**Methods:**

Computer-aided molecular docking and molecular dynamics (MD) simulations accompanied by in vitro testing were used to mine novel, pharmacologically active chemical entities from Egyptian propolis to combat Type 2 DM. Glide docking was utilized for a structure-based virtual screening of the largest in-house library of Egyptian propolis metabolites gathered from literature, in addition to GC–MS analysis of the propolis sample under investigation. Thereafter, combination analysis by means of fixed-ratio combinations of acarbose with propolis and the top chosen propolis-derived phytoligand was implemented.

**Results:**

Aucubin, identified for the first time in propolis worldwide and kaempferol were the most promising virtual hits. Subsequent in vitro α-amylase inhibitory assay demonstrated the ability of these hits to significantly inhibit the enzyme in a dose-dependent manner with an IC_50_ of 2.37 ± 0.02 mM and 4.84 ± 0.14 mM, respectively. The binary combination of acarbose with each of propolis and kaempferol displayed maximal synergy at lower effect levels. Molecular docking and MD simulations revealed a cooperative binding mode between kaempferol and acarbose within the active site.

**Conclusion:**

The suggested strategy seems imperative to ensure a steady supply of new therapeutic entities sourced from Egyptian propolis to regress the development of DM. Further pharmacological in vivo investigations are required to confirm the potent antidiabetic potential of the studied combination.

**Supplementary Information:**

The online version contains supplementary material available at 10.1186/s12906-024-04348-x.

## Background

Diabetes mellitus (DM) affects nearly one in ten adults worldwide. Amongst the different types of DM, Type 2 DM is the most prevalent; greater than 95% of people inflicted with diabetes are Type 2 diabetics [1]. In contrast to Type 1 DM, Type 2 is characterized by the body’s inability to effectively utilize insulin (insulin resistance). Despite that, hyperglycemia occurs as a result of the body’s resistance to insulin [2]. This, in turn, may result in the rise of a myriad of disorders. Besides nephropathy and retinopathy, which arise due to microvascular complications, it is well-established that DM’s macrovascular complications make it a risk factor for several cardiovascular diseases such as coronary artery disease and ischemic strokes [3, 4]. Although current management plans are numerous and varied, their main drawback, unwanted side effects, persists. Side effect-free management remains a true challenge [5].

A key carbohydrate-hydrolysing enzyme, α-amylase, is the target in one of the therapeutic approaches employed in the management of Type 2 DM. By means of α-amylase, starch, the complex dietary polysaccharide, is metabolized into simpler saccharides. Thereafter, the simple saccharides are subsequently metabolized into the readily absorbable glucose leading to an elevated postprandial blood glucose level. Through inhibition of α-amylase, hydrolysis of starch is retarded and consequently, the once-familiar spike in postprandial blood glucose level is now dampened [6, 7].

Acarbose (Glucobay®), is a drug acting through this pathway via its inhibitory action on the carbohydrates-hydrolysing enzyme, α-amylase. In addition to acarbose’s role in diabetics, according to the American Association of Clinical Endocrinology (AACE) Clinical Practice Guideline, it can be used in the prevention of the progression of prediabetics to Type 2 DM [8]. Moreover, the International Diabetes Federation recommends considering acarbose for the prevention of diabetes in at-risk individuals who fail to achieve the glucose tolerance target by means of lifestyle interventions [9]. However, the established dose of acarbose for managing postprandial blood glucose level is often accompanied by undesirable gastrointestinal side effects such as flatulence, diarrhea, and abdominal pain. In a study encompassing 714 medicated patients, almost one in five (19%) participants discontinued acarbose due to the aforementioned side effects [10]. Additionally, more serious side effects in the form of hepatic injuries such as jaundice and hepatitis have been noted through post-marketing adverse event reports [11]. Moreover, acarbose has also been listed in the FDA’s Drug-Induced Liver Injury Severity and Toxicity Dataset [12, 13]. It is therefore crucial to find a means to circumvent these troublesome side effects.

Bee propolis (bee glue) is a resinous, natural, complex product of honeybees (*Apis meliffera* L.). It is an intricate blend of exudates of multifloral origin [14]. On a chemical level, a fusion of resins along with wax, essential oils, balsams, phenolic compounds such as flavonoids, aromatic acids and their esters, and pollen grains amidst other constituents make up the concoction that is propolis [15]. The exact fabric of propolis is inconsistent and varies greatly between nonidentical samples [16] based on numerous factors including: available surrounding vegetation, climate, collection time [17], and the cultivating bee’s subspecies [18]. To further illustrate this point, European propolis has been demonstrated to contain phenolics as flavonoid agylcones, phenolic acids and their corresponding esters. Whereas Brazilian propolis is more often characterized by the presence of prenylated p-coumaric acid and acetophenone derivatives. Other prenylated derivatives such as those of benzophenone exist in propolis and are more often seen in Cuban propolis [19]. Propolis’ biological applications are only as diverse as its rich and disparate chemical profiles. The variable nature of propolis’ bioactive constituents provides insight into the multifarious biological effects that different propolis samples exhibit. These activities span an expansive list and include anti-inflammatory, antibacterial, antifungal [20], antiviral [21], antitumor [22], antioxidant [23], and antidiabetic activity [24].

To understand the biological activity of propolis, it is vital to first characterize the sample through investigation of its chemical composition. A variety of analytical approaches have been employed in the past including spectrophotometric techniques such as infrared (IR) [25], nuclear magnetic resonance (NMR) [26], and ultraviolet (UV) [27] spectrophotometry. Chromatographic techniques such as high‐performance liquid chromatography (HPLC) [28], high‐performance thin layer chromatography (HPTLC) [29], and Gas Chromatography (GC) [30] have also been utilized. In this study, GC coupled with mass spectrometry (GC–MS) has been adopted to identify and quantify phytoconstituents following derivatization. As demonstrated by Greenaway et al*.* [31], GC–MS allows for the rapid determination of over a hundred compounds in a propolis sample.

Besides searching for new and safer alternative antidiabetic agents, another approach to curb the vexing side effects brought on by acarbose is to lower its problematic dose, all while retaining the same overall therapeutic efficacy. This objective can be accomplished through combination therapy; the concomitant use of multiple agents is commonly adopted and firmly set in most challenging diseases. The main concept behind combination therapy is that superior therapeutic outcomes can be realised via synergistic drug combinations. In that, an increase in the efficacy of the treatment’s effect is observed or a decrease in the drugs’ doses and consequently reduced toxicity is achieved. In conditions where the evolution of drug resistance is feasible, combination therapy provides an answer by slowing down or eliminating the development of the aforementioned resistance. Computational analysis of multidrug combinations can provide insight into the drug-drug interaction present. In that, it is possible to discern the synergistic, additive, or antagonistic nature of a combination [32].

In this study, an Egyptian propolis sample was phytochemically investigated by means of GC–MS. An in-house library of compounds reported in Egyptian propolis was compiled from the sample under investigation, alongside an extensive literature review spanning publications published from 1997 to date. In silico docking was thereafter executed to determine the compounds with the highest affinity to α-amylase with the hope of developing new lead compounds. In vitro α-amylase assay was carried out for propolis and the top ten in silico hits. Soon after, combination analysis of acarbose with propolis was carried out to evaluate the nature and extent of the multidrug regimen. To more sharply define the drug combination, the combination of acarbose with the most potent phytoconstituent resulting from the in vitro assay was then studied. Moreover, the stability of the resulting complexes was analyzed using molecular dynamics.

## Methods

### Chemicals and reagents

3,5-dinitrosalicylic acid (DNS), acarbose, chlorogenic acid, N,O-bis(trimethylsilyl)trifluoroacetamide (BSTFA), potato starch, pyridine, quercetin, quercetin-7-methyl ether, rosmarinic acid, and α-amylase from porcine pancreas (A3176) were procured from Sigma-Aldrich (Germany). Aucubin, catechin, kaempferol, luteolin, myricetin, and quercetin-3-methyl ether were obtained from Indofine Chemical Company, Inc. (USA). Analytical purity grade solvents were used throughout the study.

### Propolis samples collection and preparation

A raw propolis sample (Fig. S1) was collected from an apiary located in Kafr El Sheikh, Egypt according to relevant guidelines and regulations.

Prior to analysis, the propolis sample was stored in the dark at ambient temperature. An aliquot of (100g) of finely pulverized Egyptian propolis sample was extracted using 1 L of 95% (v/v) ethanol by sonication for 1 h at 40 °C followed by overnight maceration. The extract was subsequently filtered, and the resulting filtrate was evaporated under reduced pressure using a rotary evaporator and kept refrigerated at 5 ℃ until further use.

### Gas chromatography‑mass spectrometry (GC–MS) analysis

#### Propolis sample solution preparation and derivatization

Silylation of propolis sample was performed on the previously prepared dry extract. According to the method reported by Popova et al*.* [33], 5 mg sample was added to 50µL of dry pyridine and 75µL of BSTFA. This mixture was then heated to 80°C for 30 min.

#### Chromatographic parameters and conditions

Chemical constituents were analyzed and determined by GC–MS instrumentation by injection of a 1µL diluted (1:10 hexane, v/v) derivatized propolis sample at a split ratio of 1:10 into a TRACE GC Ultra Gas Chromatograph (THERMO Scientific Corp., USA) partnered with a Thermo mass spectrometer detector (ISQ Single Quadrupole Mass Spectrometer). The system was equipped with a TR-5 MS column (30m × 0.32mm i.d., 0.25μm film thickness) and helium as the carrier gas was used at a flow rate of 1.0mL/min. The temperature program was initially set at 60°C for 1 min, increasing at a rate of 4°C/min to finally reach 240°C which was also held for 1 min. The sample injector and detector line were fixed at 210°C. Mass spectra were utilized by electron ionization (EI) at 70eV, and the spectral range spanned 40-450m/z. AMDIS software (www.amdis.net) was employed for spectral deconvolution, while NIST and Wiley mass spectral databases were used for the identification of compounds by comparing both the retention index (relative to n-alkanes C8-C22) and mass spectra to reference standards.

### Compilation of the largest in-house database of Egyptian propolis

An in-house database comprised of 378 phytoconstituents (Sheet S1) was constructed from the sample under investigation, in addition to an exhaustive literature review of Egyptian propolis. The review included publications spanning a 25 year period (from 1997 to date). It is considered as the largest in-house database of Egyptian propolis.

### Molecular docking of Egyptian propolis phytoligands

Molecular docking studies of α-amylase inhibitors were performed using both Schrödinger Maestro molecular modeling Suite (Schrodinger, LLC, New York) and AutoDock Vina [34] via the CB-Dock2 server [35] (https://cadd.labshare.cn/cb-dock2/php/index.php) on the in-house propolis library.

To determine the three-dimensional crystal structure that would be used in the in silico study of this work, three pancreatic α-amylase crystalline structures were downloaded from the RCSB protein data bank (PDB) and were comparatively evaluated. The crystal structures chosen included human pancreatic α-amylase co-crystallized with the flavonoid, myricetin (PDB ID: 4GQR) and with the pseudo-hexasaccharide, acarviostatin I03 (PDB ID: 3OLD), respectively. Additionally, porcine pancreatic α-amylase co-crystallized with the pseudo-tetrasaccharide, acarbose (PDB ID: 1OSE) was also included.

#### Preparation of protein structures

Initially, the enzymes were retrieved in .pdb format and then further optimized prior to execution of docking using the protein preparation wizard in Schrödinger Maestro. This entailed several key modifications including assigning bond orders, hydrogen atoms, deletion of water molecules beyond 5.00 A, and the removal of all heteroatoms, other than the Ca^+^ and Cl^−^ ions. Furthermore, disulfide bonds and zero-order bonds to metals were created. Subsequently, energy states were generated at pH 7 with a range of ± 3 and the lowest energy state generated was chosen. Finally, the protein’s hydrogen bond assignment was optimized using PROPKA at pH 7 and the overall structure underwent energy minimization using the OPLS3 forcefield algorithm until attaining a relative mean standard deviation (RMSD) above 0.30°A as compared to the crystal structure. The resultant protein was thereafter used in the docking process.

#### Ligand preparation

By the same token, preparation and optimization of ligand molecules were carried out by means of LigPrep module in Schrödinger Maestro. This involved generation of various tautomers, stereoisomers and all possible protonation states present at the physiological pH range (pH 7 ± 2) using Epik. In addition, the ligands were also desalted and compounds with defined chirality were retained; in contrast, those with unspecified chirality were set to generate a maximum of 32 different stereoisomers. These structures underwent optimization via OPLS3 forcefield.

#### Grid preparation and docking

Grid generation was the final step performed before the resulting structures were subjected to docking studies. A receptor grid was generated by selecting the box enclosing the centroid of the complexed ligandin Glide.

For docking calculations, extra-precision (XP) mode was selected. The resulting intermolecular interactions with the highest docking scores were then analyzed and visualized using Maestro interface, UCSF ChimeraX [36] version 1.4 and BIOVIA Discovery Studio Visualizer v21. For validation of the molecular docking study, the cocrystallized ligand, myricetin in the 4GQR structure was self-docked and the RMSD value between the natural binding pose and the docking pose was measured.

#### Validation of docking process

Forty molecules with previously confirmed α-amylase inhibitory activity made up the validation set (Table S1) employed herein. The validation set, alongside 1000 decoys were docked against the aforementioned crystalline structures; this was done to evaluate the crystalline structures’ ability to differentiate between the α-amylase inhibitors and the decoys. The docking process was carried out as mentioned in Sect. 2.5.3. The accuracy of GLIDE docking was assessed through use of the GLIDE enrichment calculator, where several parameters including ROC, AUC-ROC, BEDROC (α = 8, 20, and 160.9), EF (2%, 5%, and 10%), sensitivity, and specificity were determined and compared.

Furthermore, the root mean square deviation (RMSD) for each crystalline structure was sought to further validate the docking protocol. This was accomplished by utilizing the pose selection method to re-dock the co-crystallized ligand into its designated binding site in each respective crystalline structure. Thereafter, the docked pose was compared to the crystal structure’s pose and the RMSD was calculated.

### In vitro pancreatic α-amylase inhibitory assay

With reference to the established technique [37], the assay was carried out quantitatively with minor adjustments. Initially, 500µL test solution was preincubated with 500µL α-amylase solution (0.55 unit/mL) at 25°C for 10 min. The α-amylase was prepared in a buffer solution (0.02M sodium phosphate pH 6.9 with 0.006M NaCl). Afterwards, 500µL of 1% w/v starch in buffer solution was mixed in and left to incubate for 10 min at 25°C. To bring the reaction to a halt, 1mL dinitrosalicylic acid (DNS) was introduced and the mixture was placed in a boiling water bath for 3.5 min. After cooling to room temperature, the mixture was then diluted using 10mL distilled water and absorbance was measured using a Laxco spectrophotometer (α1502, Laxco Inc., USA) at λ 540nm. Blanks used throughout the experiment were made by adding pure buffer instead of α-amylase solution. Acarbose was used as positive control. To calculate the pancreatic α-amylase inhibitory activity, the following equation was used:

$$\%\;Inhibtion=100\times\left[1-\frac{Abs_{540nm}^{Extract}-{Abs}_{540nm}^{Positive\;control}}{{Abs}_{540nm}^{Negative\;control}-{Abs}_{540nm}^{Positive\;control}}\right]$$

### Combination analysis

CompuSyn software (www.combosyn.com) was employed to assess the nature of the test substance-acarbose interaction. Propolis and kaempferol were each individually paired with acarbose and investigation was carried out using four different analysis methodologies. These include: median effect, isobolographic, combination index, and lastly, dose reduction index analyses.

Data input included each inhibitors’ independent activity and the summation of both inhibitors’ activity when used in conjunction of each other. For combination assay, the inhibitors were mixed at five different concentration levels to give a final concentration equal to the concentration required to achieve 10, 30, 50, 70, and 90% inhibition of α-amylase when used independently. α-amylase inhibitory activity assay was conducted as per Sect. 2.6.

#### Median-effect analysis

This method of analysis relies on its namesake equation, the median-effect equation which stems from the general mass-action law principle. Each inhibitor’s sigmoidal dose–effect plot was individually generated in addition to their respective combination. Thereafter, the plot was transfigured linearly into median-effect plots. The median-effect equation is described as [38]:

$$\frac{{f}_{a}}{{f}_{u}}={\left(\frac{D}{{D}_{m}}\right)}^{m}$$

D represents the dose of the inhibitor, ${D}_{m}$ is the inhibitor’s dose causing a decrease of 50% in the enzyme’s activity, ${f}_{a}$ is the fraction affected by dose $D$, ${f}_{u}$ is the unaffected fraction (${f}_{u}$ = 1 – ${f}_{a}$), and $m$ the curve’s sigmoidicity coefficient. Through logarithmic transformation, the equation becomes:

$$Log\left(\frac{{f}_{a}}{{f}_{u}}\right)=mlog\left(D\right)-mlog({D}_{m})$$

This, in turn, results in the median-effect plot. A plot where $y=log(\frac{{f}_{a}}{{f}_{u}})$ against $x=log(D)$ with $m$ being the slope and $log({D}_{m})$, the x-intercept. ${D}_{m}$ and $m$ are obtained from the median-effect plot. To determine the data’s conformity to the general mass-action principle, the linear correlation coefficient (r) of the plot is assessed.

#### Isobolographic analysis

Using each inhibitor’s individual effect, this method of analysis is employed to assess the combined effect of two inhibitors. A graph, wherein the x- and y- axes represent the doses of inhibitors A and B, respectively, is plotted. The doses, a and b denote the dose of inhibitor A and B, respectively, resulting in the same efficacy when used individually. (e.g.: At $x$% inhibition, a = ${C}_{A,x}$ and b = ${C}_{B,x}$). To assess the combined effects of inhibitors A and B, an additive line must first be established. This is accomplished by connecting two points of the same efficacy (e.g.: connecting a with b). Based on the respective position of the combination data points relative to the additive line, the nature of the interaction can be determined; synergism, addition, or antagonism are indicated by data points lying below, on, or above the additive line, respectively [39].

#### Combination index analysis

The combination index (CI) quantitatively evaluates the type of interaction between two inhibitors at a series of inhibition levels. CI is calculated by means of the following equation [38]:

$$CI=\frac{{C}_{A,x}}{{IC}_{x, A}}+\frac{{C}_{B,x}}{{IC}_{x,B}}$$

Where ${IC}_{x, A}$ and ${IC}_{x, B}$ are the doses of inhibitors A and B, respectively, required to produce $x$% inhibition when each inhibitor is used individually. Whereas ${C}_{A,x}$ and ${C}_{B,x}$ are the doses of inhibitors A and B, respectively, required to produce $x$% inhibition when a binary mixture is used. Based on the resulting CI, the nature of the inhibitors’ interaction can be concluded. A CI lower than, equal to, or greater than 1 is indicative of synergism, addition, or antagonism, respectively. Alternatively, the interaction present between the two inhibitors can be deduced via the combination index (${f}_{a}$-CI) plot [40].

#### Dose reduction index analysis

The dose reduction index (DRI) was calculated for synergistic binary combinations to measure the fold decrease in the dose of each inhibitor at a given effect level, relative to the dose of each inhibitor when used solitarily resulting in the same effect. DRI can be calculated as follows [32]:

$$DRI=\frac{{IC}_{50}\lbrack solitary\;inhibitor\rbrack}{{IC}_{50}\lbrack inhibitor\;in\;combination\rbrack}$$

DRI values > 1 are favored, reflecting a decrease in dose while maintaining or enhancing the inhibitor’s efficacy [41].

### Molecular dynamics simulations and trajectory analysis

Molecular dynamics (MD) simulations of α-amylase with acarbose, α-amylase with kaempferol, and α-amylase with acarbose and kaempferol co-ligand complexes obtained by molecular docking were performed with Gromacs v2020.1 [42]. MD input files were created with the CHARMM-GUI [43] server's Solution Builder tool (https://charmm-gui.org/?doc=input/solution). Protein–ligand complexes were solvated with the TIP3 water model and neutralized by adding 0.15 M KCl using the Monte Carlo method. Protein–ligand topology files were created with AMBER FF99SB [44] force fields. For MD simulation, it was equilibrated with the Nose–Hoover thermostat and Parrinello-Rahman barostat methods at 303.15 K and 1.0 atm pressure. Bond constraints were made with hydrogen bonds according to the LINCS algorithm. MD simulation was performed under periodic boundary conditions for 150 ns. For MD trajectory analysis, root mean square deviation (RMSD) was created with gmx rms script, graphics were created with Grace-5.1.2, and MD animation videos were created with PyMOL Molecular Graphics System v2.4.1. Binding free energy Molecular Mechanics Poisson-Boltzmann Surface Area (MM-PBSA) calculations were calculated from 1500 frames recorded for 150 ns with the gmx_MMPBSA [45] tool.

## Results and discussion

### GC–MS analysis of silylated bioactive propolis sample

Owing to the chemical diversity between propolis samples, standardization of propolis is critical in order to guarantee its chemical consistency and thereafter, consistent efficacy. Therefore, it has been noted that for one to be ascertain of the biological activity, we must in turn first chemically characterize propolis. One of the most frequently employed methods in the chemical analyses of propolis is GC–MS [19, 46].

Silylation is a crucial step required prior to analysis of the relatively non-volatile propolis components to assist in their separation. Trimethylsilyl (TMS) derivatives are more volatile, more thermotolerant and less polar than their corresponding parent compound.

Although other analytical techniques not requiring prior extensive sample preparation can be employed in the separation of propolis constituents, the unequivocal separation capabilities and resolution provided by capillary GC and the invaluable structural data brought forth by EIMS make it worth the additional derivatization step. Numerous silylating agents can be used with propolis, each with their own set of advantages and disadvantages. In the current study, the examined propolis sample was subjected to BSTFA as the silylating agent as it shows high reactivity with all common polar functional groups.

**Fig. 1:**
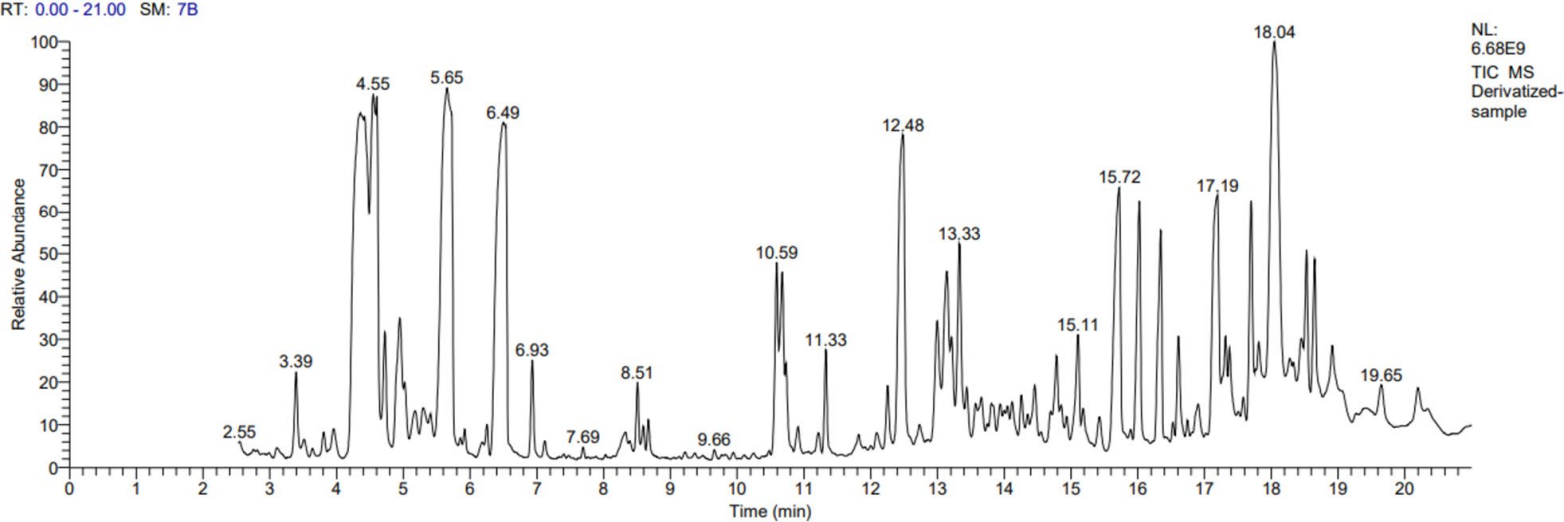
Total ion chromatogram of derivatized ethanolic extract of Egyptian propolis sample

The GC–MS analysis runtime lasted 21 min and the resulting TIC chromatogram (Fig. 1) revealed that the selected Egyptian propolis sample contained a total of 78 compounds. Of the separated derivatized compounds, 27 compounds (Table 1) were subsequently identified through comparison of their spectral data with Wiley and NIST mass spectral databases. The retention time (min.), compound name, phytochemical class, % TIC by normalization, molecular masses, and library used in identification of the compounds are described in Table 1.

**Table 1:** Chemical composition of derivatized ethanolic extract of Egyptian propolis sample as assessed by GC-EI-MS

**Peak No**	**t**_**R**_** (min.)**	**Compound Name**	**Phytochemical Class**	**TIC %**	**MWT**	**CAS**	**MF**
1	3.11	α-Arabinopyranose^i,a^	Sugar	0.15	150.13	608–45-7	C_5_H_10_O_5_
2	3.39	Cinnamyl alcohol^i,a^	Aromatic alcohol	0.81	134.17	4407–36-7	C_9_H_10_O
3	3.81	α-Curcumene^iii,a^	Aromatic Hydrocarbon	0.21	202.33	644–30-4	C_15_H_22_
4	4.36	D-Fructose^iii,a^	Sugar	12.11	180.16	7660–25-5	C_6_H_12_O_6_
5	4.95	Myo-inositol^iii^	Sugar	2.24	180.16	6917–35-7	C_6_H_12_O_6_
6	5.17	D-Mannitol^i^	Sugar	0.31	182.17	69–65-8	C_6_H_14_O_6_
7	5.3	Talopyranose^i,a^	Sugar	0.35	180.16		C_6_H_12_O_6_
8	5.66	D-Glucopyranose^i^	Sugar	9.31	180.16	2280–44-6	C_6_H_12_O_6_
9	5.86	D-Ribopyranose^i,a^	Sugar	0.06	150.13	10257–32-6	C_5_H_10_O_5_
10	6.18	D-Gluconic acid^i^	Sugar	0.14	196.16	526–95-4	C_6_H_12_O_7_
11	6.25	Galactopyranose^iii,a^	Sugar	0.23	180.16	10257–28-0	C_6_H_12_O_6_
12	6.5	Hexopyranose^iv,a^	Sugar	8.86	180.16	42752–07-8	C_6_H_12_O_6_
13	7.69	Gallic acid^iii^	Phenolic Compound	0.11	170.12	149–91-7	C_7_H_6_O_5_
14	8.32	Palmitic Acid^iii^	Fatty Acid	0.43	256.43	57–10-3	C_16_H_32_O_2_
15	10.59	Isoferulic acid^i^	Phenolic Compound	1.61	194.18	537–73-5	C_10_H_10_O_4_
16	10.91	Linoleic acid^i^	Fatty Acid	0.24	280.45	60–33-3	C_18_H_32_O_2_
17	11.22	α–Linolenic acid^iii,a^	Fatty Acid	0.22	278.44	463–40-1	C_18_H_30_O_2_
18	11.82	Aucubin^i,a,b^	Iridoid glycoside	0.25	346.33	479–98-1	C_15_H_22_O_9_
19	12.48	Sucrose^i^	Sugar	5.07	342.3	57–50-1	C_12_H_22_O_11_
20	13	Maltose^i,a^	Sugar	1.4	342.3	200–716-5	C_12_H_22_O_11_
21	13.33	D-( +)-Cellobiose^i,a^	Sugar	1.65	342.3	528–50-7	C_12_H_22_O_11_
22	14.12	D-( +)-Turanose^i,a^	Sugar	0.31	342.3	547–25-1	C_12_H_22_O_11_
23	14.26	α–D-Lactose^i,a^	Sugar	0.33	342.3	14641–93-1	C_12_H_22_O_11_
24	15.11	trans-methyl 2-methyl-3-(2-hydroxyphenyl)-3,4-dihydro-1(2H)-isoquinoline-4-carboxylate ^iv,a^	Aromatic Ester	1.17	297.35	-	C_18_H_19_NO_3_
25	15.72	2,4,4'-Trihydroxychalcone^iv^	Phenolic Compound	4.09	256.26	83616–07-3	C_15_H_12_O_4_
26	17.18	Genistein^i^	Flavonoids	3.53	270.24	446–72-0	C_15_H_10_O_5_
27	18.04	Chrysin^i^	Flavonoids	7.08	254.24	480–40-0	C_15_H_10_O_4_

^i^^, ii, iii, iv^ Identified using NIST mainlib, NIST_msms, NIST replib and Wiley Registry 8e spectral libraries, respectively

^a^Reported for the first time in Egyptian propolis

^b^ Reported for the first time in worldwide propolis

The overall chemical composition of propolis varies greatly from one sample to another. Thus, it has been documented that a plethora of compounds belonging to a diverse list of classes may be found in each propolis sample. These classes include alcohols, aldehydes, aliphatic acids and their esters, amino acids, aromatic acids and their ester, ethers, fatty acids, flavonoids, hydrocarbon esters, ketones, steroids, sugars and terpenoids amongst others [17]. Of the 27 compounds identified by the aforementioned means, 12 of them are well-documented in Egyptian and worldwide propolis. These include d-glucopyranose [47–49] (10.6%); chrysin [46–48, 50–59] (8.1%); sucrose [47, 48] (5.8%); 1-[4-hydroxyphenyl]-3-[2,4-dihydroxyphenyl]-2-propen-1-one [57] (4.7%); genistein [53, 54, 56–58, 60] (4%); myo-inositol [50, 61] (2.5%); isoferulic acid [46, 50, 52, 58–60, 62] (1.8%); palmitic acid [46–48, 50–52, 57, 58, 60, 62–65] (0.5%); d-mannitol [47, 48] (0.4%); linoleic acid [46, 50, 52, 58] (0.3%); d-gluconic acid [50, 52, 63] (0.2%); and gallic acid [52, 58, 66] (0.1%).

On the other hand, 15 of the 27 identified compounds are reported for the first time in Egyptian propolis. Uniquely, aucubin, one of these 27 compounds is reported for the first time in propolis worldwide. To further confirm the presence of aucubin in the sample, the spectra and retention time was compared with that of standard reference aucubin.

The stereotypical chemical profile of Egyptian propolis is distinguishable by its rich content of flavonoids and phenolic acids and their esters [50].This, however, is not always the case, as Hegazi et al*.* previously reported on Egyptian propolis rich in triterpenoids which made up 17.3% of the total composition [63]. Hegazi *et. al* also characterized an Egyptian propolis sample by its benzofuran lignans content (13.5%) in 2007 [52]. Moreover, in 2014, Morsy et al*.* identified an Egyptian propolis sample rich in fatty acids [65]. In this study, it has been found that the sample's chemical profile bears a strong resemblance to that of Maltese propolis as reported by Popova et al. [33], being that the most abundant compounds present are sugars. The source of these sugars reiterate the long-standing hypothesis that plant mucilage could be another source bees rely on for propolis [33]. This is not the first time that carbohydrate and sugar rich Egyptian propolis samples were studied, as this has been noted by Christov et al*.* as early as 1998 [47].

Besides sugars, the remaining compounds in the studied sample exhibited great diversity in chemical nature, these compounds belonged to the following classes: aromatic hydrocarbons, fatty acids, phenolic compounds, aromatic esters, flavonoids, glycosides, and aromatic alcohols.

Contrastingly, the top four most highly abundant constituents did not all vary in chemical nature; with the most abundant constituent being d-fructose (13.77%), closely followed by d-glucopyranose (10.59%), hexopyranose, (10.08%) and lastly, chrysin (8.05%). Their EI-MS spectra are presented in Figure S2. The fragmentation patterns of the four major compounds are highlighted in Table S2, together with their detailed characterization and fragmentation schemes (Figures S3-S6).

### Molecular virtual screening of Egyptian propolis phytoligands

Virtual screening (VS) is a computational strategy that is used in drug discovery [67]. Its main application is the identification of top-hit compounds and optimization of lead compounds. Compared with other traditional experimental screening techniques, VS has the main advantage of being fast and cost effective [68, 69]. VS can be classified according to the method of screening into structure-based and ligand-based methods.

Structure-based virtual screening includes molecular docking which is the most widely used method [70]. Molecular docking models the interaction occurring between the test molecule and the target protein at an atomic level [71]. As for Ligand-based virtual screening, this includes methods such as pharmacophore modelling and Quantitative Structure Activity Relationship (QSAR). This method of screening relies on the presence of a set of active ligand molecules and it correlates their activity to structural information [72].

Docking studies may be done without specifying the binding site within the protein, this is called blind docking. However, in order to increase the efficiency of docking, it is recommended to locate and specify the binding site. This can be done by analyzing the structure of the target protein crystallized with a known ligand.

For centuries, people have relied on products of natural origin in the treatment and prevention of a myriad of diseases. However, natural products are not suited for high throughput screening drug discovery due to several reasons. To start, natural compounds are found in minute amounts and following their extraction and purification, these compounds are obtained in very limited quantities. Additionally, natural compounds exhibit high structural complexity and therefore, their synthesis is an incredibly difficult task. Therefore, structure-based drug discovery can be implemented using a library of pure natural compounds. This will in turn decrease the time, resources and effort wasted that would otherwise be used in in vitro screening.

#### Comparative evaluation of the three enzyme crystal structures and validation of docking process

To select the most suitable enzyme crystal structure and to validate the docking procedure used in this study, two distinct approaches were undertaken. The first, made use of the RMSD value which was obtained through use of the ligand co-crystallized with each crystal structure. As highlighted in Table 2, for the three α-amylase crystal structures: 4GQR (co-crystallized with myricetin), 3OLD (co-crystallized with acarviostatin), and 1OSE (co-crystallized with acarbose), the RMSD value was less than 1 Å, which reflects high docking accuracy. While 3OLD and 1OSE had nearly similar RMSD values of 0.607 and 0.682, respectively, 4GQR stood out with the lowest RMSD of the three enzymes, with an RMSD value of 0.449 Å.

**Table 2:** Validation parameters of the molecular docking of three pancreatic α-amylase crystalline structures

Validation Parameter	4GQR	3OLD	1OSE
RMSD^a^	0.449	0.607	0.682
AUC-ROC	0.999	0.999	0.999
EF (2%)	24	24	24
EF (5%)	20	20	20
EF (10%)	10	10	10
RIE	13.62	13.62	13.62
BEDROC (*α* = 8)	1	1	1
BEDROC (*α* = 20)	1	1	1
BEDROC (*α* = 160.9)	1	1	1
Ranked actives^b^	43	43	43
Approximate Sensitivity	1	1	1
Specificity	1	1	1

^a^ RMSD values were calculated for each enzyme using the enzyme’s crystalline structure and its respective co-crystallized ligand

^b^ Ranked actives correspond to the number of actives recovered from the employed validation set

Moreover, a validation set comprised of 40 compounds with documented α-amylase inhibitory activity (Table S1) and 1000 decoys was docked. Thereafter, the specificity and sensitivity of each crystal structure was analyzed to evaluate which crystal structure has better predictive potential. Further, ROCs were plotted and parameters including AUC-ROC, BEDROC (*α* = 8, 20, and 160.9), and EF (2%, 5%, and 10%) were calculated.

Through ROC plots, all three enzyme crystal structures demonstrated the ability to separate α-amylase inhibitors from inactive decoys, with all crystal structures yielding a specificity of unity. Similarly, all three structures had very high sensitivity with an approximate sensitivity value of one. Likewise, the three structures matched in AUC-ROC value which was observed to be 0.999. AUC-ROC was employed to measure how highly a random active is ranked in comparison to a random decoy [73]. Furthermore, the three crystal structures were found to be in agreement in all EF (2%, 5% and 10%) values. The EF values express the ability of the enzyme to pick an active from a seeded random set. The EF percentage denotes that the top respective percentage from the total set is considered. For EF (2%), EF (5%), and EF (10%), the maximum attainable enrichment factors are 50, 20, and 10, respectively [74].

Lastly, BEDROC is used to evaluate the crystal structure’s ability to discern between actives and decoys at varying tuning parameter value α [75]. Akin to all previous validation parameters sans RMSD, all three crystal structures agreed in BEDROC values.

As noted from the docking validation parameters in Table 2, all three crystal structures are identical in most aspects. However, through use of RMSD, we have established that 4GQR is the most suitable crystal structure and thus, has been employed in the docking studies to follow. Although the use of 4GQR has been reported in several studies; with some being as recent as 2023, as in a study by Mohamed et al*.* [76], the other crystal structures we evaluated were also employed in various in silico invesigations. In 2022, Vo Van et al*.* reported on the inhibitory activity of flavonoids by docking them against 1OSE [77]. Likewise, Lee et al*.* examined the effect of flavonoids using 3OLD [78].

#### Molecular virtual screening of Egyptian propolis phytoligands on 4GQR

Through use of virtual screening, prospective α-amylase inhibitors of natural origin were investigated. The present study identified potential binding affinities present between the target enzyme, α-amylase and a number of ligands. An in-house library comprised of 378 metabolites naturally occurring in Egyptian propolis was constructed and employed (Sheet S1). Numerous crystal structures of α-amylase from several source, both with and without co-crystallized ligands have been characterized and made available online. Human pancreatic α-amylase (HPA) co-crystallized with myricetin (PDB ID: 4GQR) was chosen to provide more plausible insight into in vivo binding, in addition to its high resolution of 1.20 Å.

HPA is made up of three distinct structural domains. Its active site is situated at the extremity of a triose-phosphate isomerase barrel. Therein, three critical amino acids (ASP197, GLU233 and ASP300) which are responsible for the catalysis of glycosidic bonds lie. Additionally, the presence of chloride and calcium ions is vital for catalysis of the substrate [79].

First, to test and validate the method of the molecular docking study, myricetin in the α-amylase 3D structure was re-docked, and the maximum common structure between the native binding pose and the docking pose was measured as RMSD = 0.012 Å. This value showed that the molecular docking method made successful predictions. Docking was then executed as described in Sect. 2.5 using the in-house library. Thereafter, based on the hits’ extra precision docking score, they were ranked, and the resulting top twenty hits are listed in Table S3. An array of chemical classes materialized the list including flavonoids, glycosides, and phenolic acids, amongst others. Myricetin, aucubin, and chlorogenic acid were identified as the top three hits with the highest affinity to α-amylase and lowest docking XP Gscores equal to -10.204, -10.001, and -9.421, respectively. The 2D and 3D bound conformation of the top three hits can be seen in Figure S7.

As anticipated, myricetin being the co-crystallized ligand and the top hit of screened database demonstrated the highest affinity to α-amylase and the lowest docking score. With a total of seven hydrogen bonds occurring with amino acid residues TRP59, GLN63, THR163, ASP197, HIE299, GLU233, and ASP300. Alongside the π – π stacking interaction present in ring A, a hydrophobic pocket was responsible for engulfing nearly the entirety of myricetin with the exception of ring B which was subject to a negatively charged pocket. The side chains participating in the hydrophobic interaction include TRP58, TRP59, TYR62, LEU165, PHE256, and ILE235. Whereas ASP197 and ASP300 were involved in the negatively charged interaction (Fig. S7A, B).

Similar to myricetin, aucubin formed a high number of hydrogen bond interactions with a total of six bonds occuring with amino acid residues TRP59, GLN63, ASP197, HIE299, GLU233, ASP300. It is worth noting that the amino acids participating in the hydrogen bond interactions mirror those of myricetin sans GLN63. Moreover, hydrophobic interactions were present with rings A and B engaged with TRP59, TYR62, and LEU162 (Fig. S7C, D). Remarkably, this is the first study investigating the in silico interaction between aucubin and α-amylase.

Contrastingly, chlorogenic acid’s hydrogen bond interactions amounted to only four with the amino acid residues GLN63, THR163, and GLU233. Hydrophobic interaction also played a role as a result of Van der Waals attraction with TRP58, LEU162, LEU165, and ALA198 (Fig. S7E, F).

To further validate these findings, the well-established α-amylase inhibitor, acarbose, was included as positive control. As portrayed in Figure S8A, B, acarbose exhibited interactions coinciding with those previously mentioned. Six hydrogen bonds were present between acarbose and GLN63, THR163, ASP197, GLU233, HIS299, AND ASP300 amino acid residues. Additional hydrophobic bonds with various amino acid residues such as TRP58, TRP59, TYR62, LEU162, ALA198, and PHE256 amongst others were present. Our results were comparable with previously studied α-amylase interactions by B. Kikiowo, 2020, which reported three essential amino acid residues (ASP197, GLU233 and ASP300) characterizing the active site. He further studied interactions of quercetin (3’-hydroxykaempferol) where it formed three strong H-bonds through the phenyl hydroxyl groups with the negatively charged residues ASP197 and ASP300. Alqahtani et al., 2019, explained that ASP197 acted as a catalytic nucleophile during the hydrolysis of starch as a representative to polymeric substrates [80].

By visualizing the molecular interactions of the selected top three in silico hits, we gained insight into the inner working of potential α-amylase inhibitors. However, in vitro testing is necessary to further ascertain their inhibitory activity.

### Top scoring compounds’ in vitro α-amylase inhibitory activity

**Fig. 2:**
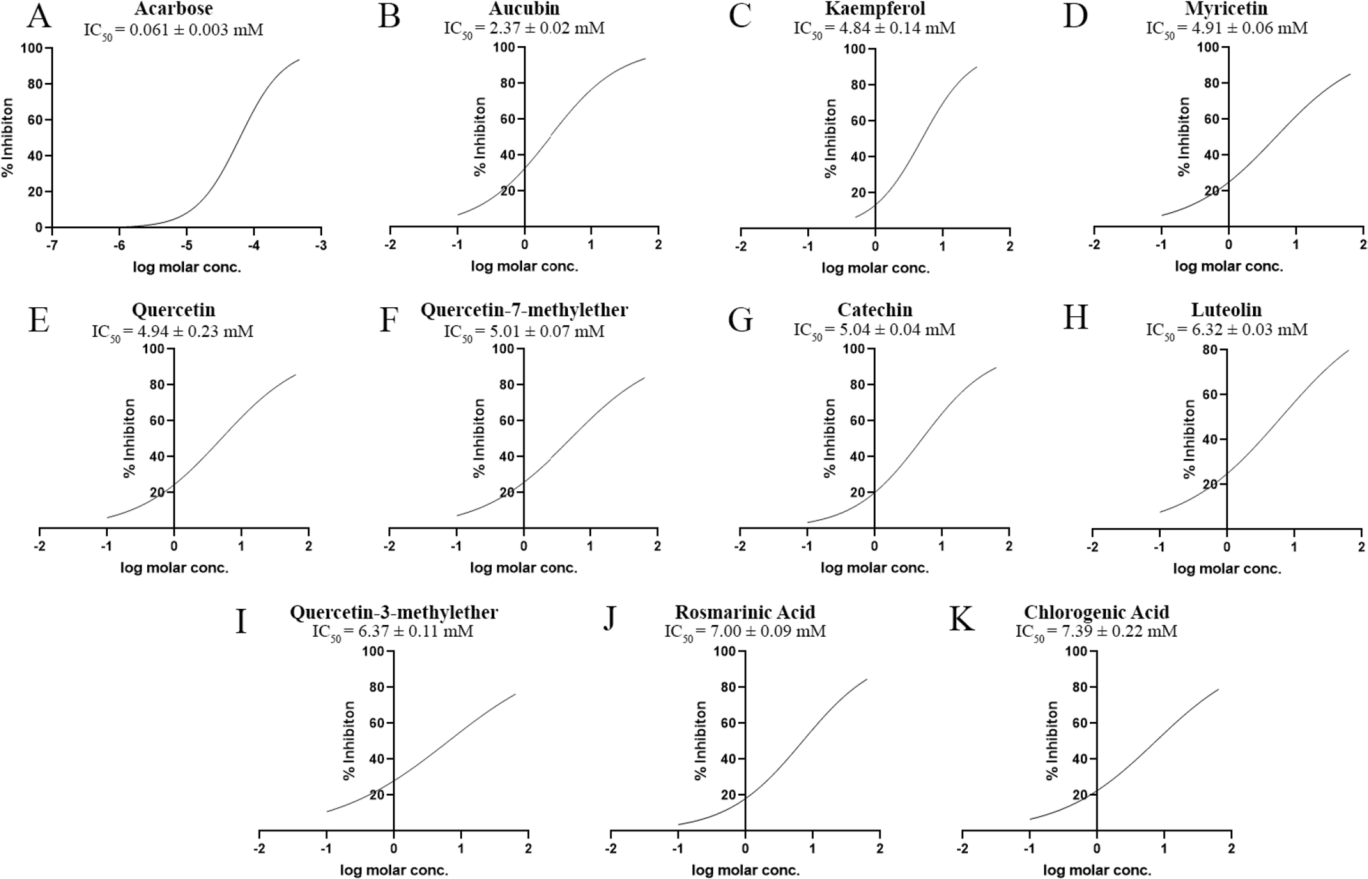
Concentration-dependent inhibition of α-amylase by acarbose (**A**) and the top in silico hits: aucubin (**B**), kaempferol (**C**), myricetin (**D**), quercetin (**E**), quercetin-7-methylether (**F**), catechin (**G**), luteolin (**H**), quercetin-3-methylether (**I**), rosmarinic acid (**J**), and chlorogenic acid (**K**)

With the aim of developing alternative α-amylase inhibitors, lead compounds from Egyptian propolis were examined. Following the promising in silico docking results, the top 10 hits' antidiabetic activity was further investigated through in vitro assay. These hits, in descending order, included myricetin, aucubin, chlorogenic acid, quercetin-7-methyl ether, quercetin, rosmarinic acid, catechin, luteolin, quercetin-3-methyl ether, and kaempferol. Their inhibitory activity was spectrophotometrically assayed at 540 nm as reported under Sect. 2.6. Acarbose, a well-established α-amylase inhibitor, was included as positive control. The concentration of each compound required to produce 50% α-amylase inhibition was determined by plotting the concentration–response curve (Fig. 2).

As illustrated in Fig. 2, all compounds demonstrated dose-dependent inhibition of α-amylase. Aucubin, an iridoid glycoside, exhibited the highest inhibitory activity with an IC_50_ of 2.367 mM. Although aucubin has been documented to possess antidiabetic activity [81], this is the first in vitro study to investigate its effect on α-amylase. It is worth noting that the only other study mentioning aucubin’s anti- α-amylase activity predicted it by employing multivariate analysis to correlate aucubin’s concentration to the crude extract overall activity without testing aucubin individually [82]. The scarce investigations could be due to the fact that despite its great antidiabetic potential, aucubin’s main drawbacks revolve around its instability, difficult isolation, and low yield [83]. At slightly greater than two-fold aucubin’s IC_50_, Kaempferol was the second most potent α-amylase inhibitor with an IC_50_ of 4.84 mM. In contrast to aucubin, Kaempferol’s in vitro anti-α-amylase activity has been previously reported with similar IC_50_ value [84], as well as in crude extracts [85].

### Study of combination therapy

Multidrug therapy has long been a lucrative option for therapeutic challenges. Through use of low-dose combinations of drugs with mutually exclusive toxicities, better treatment outcomes with lower adverse effects (as opposed to monodrug therapy) can be achieved. Insight into the nature of the drug-drug interaction (synergism, addition, antagonism) of combination therapy can be envisioned through computational analysis [32]. Going forward with the in vitro results, the inhibitory α-amylase activity of acarbose, alongside the most potent and available propolis-derived constituent, kaempferol, was investigated. Additionally, the binary combination of acarbose-propolis was also evaluated.

#### Combination therapy evaluation using median-effect analysis

**Fig. 3:**
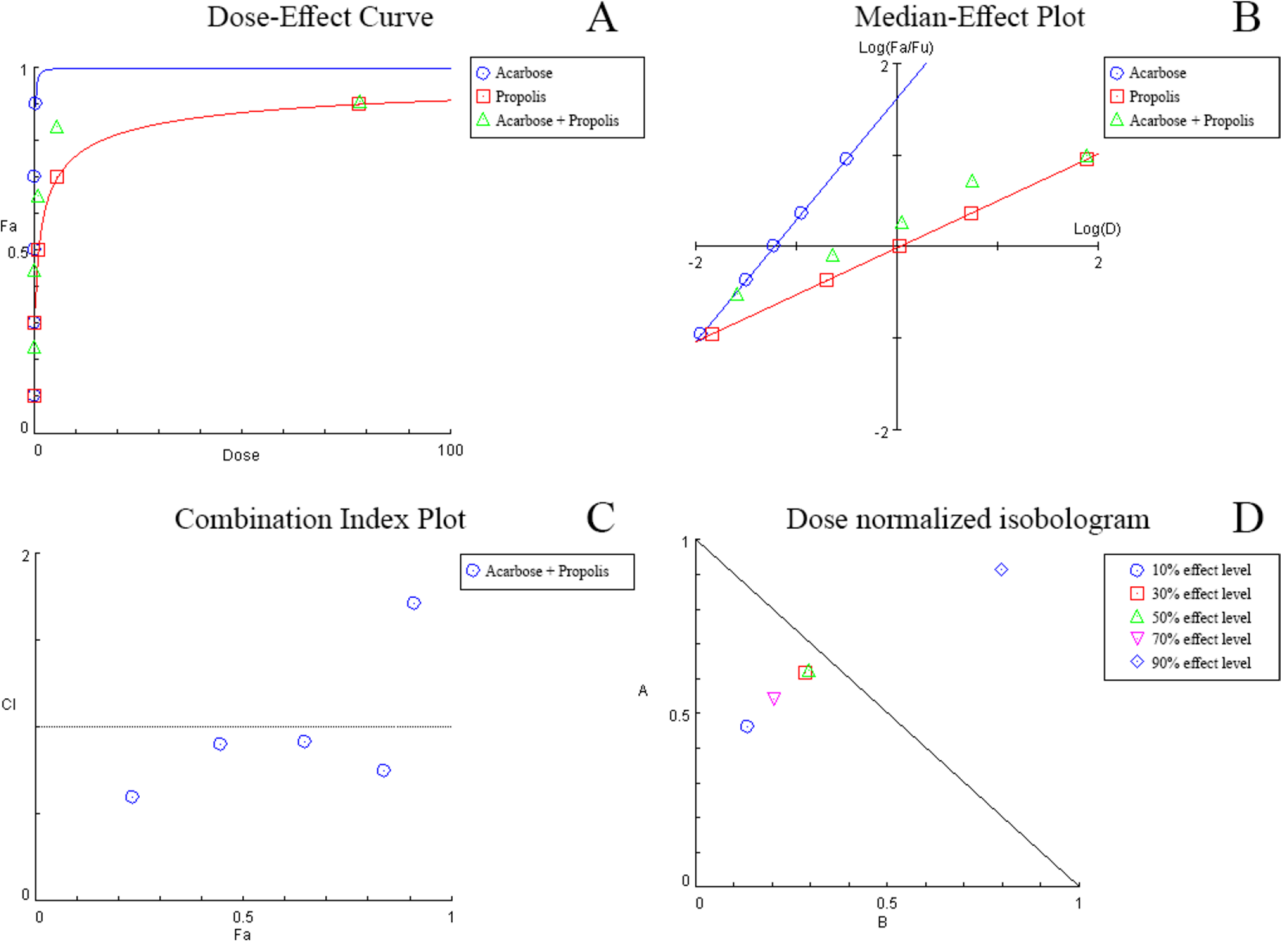
Combination analysis of acarbose and propolis showing the dose–effect curve (**A**), median-effect plot (**B**), combination index plot (**C**), and dose normalized isobologram (**D**)

**Fig. 4:**
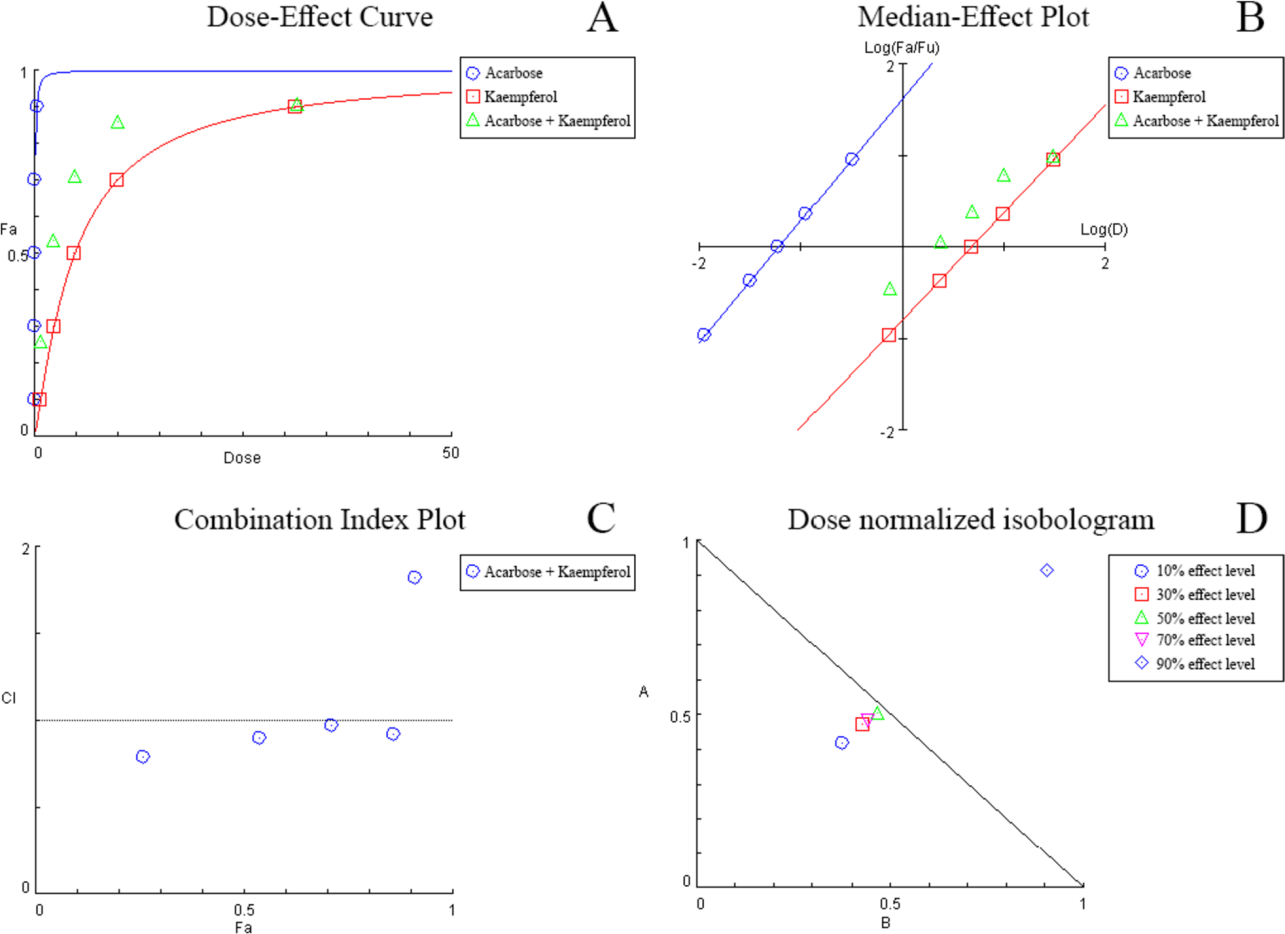
Combination analysis of acarbose and kaempferol showing the dose–effect curve (**A**), median-effect plot (**B**), combination index plot (**C**), and dose normalized isobologram (**D**)

The median-effect model, which is based on Chou’s theory [32], examines the dose-dependent effect of the inhibitors on α-amylase. A dose–effect plot is generated for each agent individually and in binary combination (Figs. 3A, B, 4A, B). Through use of CompuSyn software or via simple calculations, the parameters: ${D}_{m}$, $m$ and *r* can be obtained. ${D}_{m}$ represents the dose resulting in 50% enzymatic activity inhibition. $m$ and *r* are the slope and correlation coefficient of the plot, respectively. Table 3 summarizes the values determined for all dose–effect plots. The ${D}_{m}$ values expressed for the combination of acarbose with each of propolis and kaempferol indicate synergism at the 50% effect level, as the ${D}_{m}$ values were lower than the average of the two agents’ summed individual effect. The linearity of data in all cases was made evident through the high (≥ 0.99) *r* value.

**Table 3:** Median-effect analysis of each of acarbose, propolis, kaempferol, and their binary combinations ^a^

Drug	Dose–effect curve parameters		
	${D}_{m}$	*m*	*r*
Acarbose	0.061	1.32	1.00
Kaempferol	4.84	1.18	1.00
Propolis	1.07	0.512	1.00
Acarbose + Kaempferol	2.01	0.935	0.99
Acarbose + Propolis	0.274	0.443	0.99

^a^ ${D}_{m}$, *m*, and *r* are the antilogarithm of the x-intercept, the slope, and the correlation coefficient of the plot, respectively

#### Combination therapy evaluation using isobolographic, combination index, and dose reduction index analyses

To further discern the nature and extent of the interaction existing between the drugs, rigorous analyses methods yielding complementary information were employed. These include isobolographic, combination index, and dose reduction index analyses.

Overall, when compared to singly drug treatment, the binary combination of acarbose with either propolis or kaempferol showed a significantly greater decrease in enzymatic activity. In case of concomitant use of acarbose and propolis, synergism (CI < 1) was noted at all levels except at the 90% effect level (Table 4) and (Fig. 3C). This indicates that at lower propolis doses, the inhibitory α-amylase activity exhibited by acarbose is heightened. This was reiterated through isobolographic analysis, where the 90% effect level data point appeared above the additive line (Fig. 3D); further confirming that higher propolis doses in the combination resulted in an inferior inhibitory effect through antagonistic interaction with acarbose. Moreover, DRI analysis (Table 4) was utilized to theoretically estimate the magnitude of dose-reduction obtained owing to the synergistic nature of the combination. As such, it was estimated that to obtain 23.5% α-amylase inhibition, 0.025 mM acarbose or 0.106 mg/mL propolis would be needed. However, to attain the same inhibitory activity through binary combination, the dose would lessen by 2.16- and 7.28-fold, respectively, to be 0.012 mM acarbose and 0.015 mg/mL propolis.

**Table 4:** α-amylase fractional inhibition by the binary combination of acarbose with both propolis and kaempferol at different effect levels, their CI and DRI values ^a^

Binary combination of acarbose and propolis					
(Fa × 100) % Inhibition of combination	CI value	Dose (mM) Acarbose	Dose (mg/mL) Propolis	DRI Acarbose	DRI Propolis
23.5	0.601 (syn)	0.025	0.106	2.16	7.28
44.8	0.903 (syn)	0.052	0.710	1.62	3.50
65.1	0.920 (syn)	0.097	3.61	1.60	3.39
84	0.748 (syn)	0.212	27.3	1.84	4.88
91	1.71 (ant)	0.348	98.3	1.09	1.25
Binary combination of acarbose and kaempferol					
(Fa × 100) % Inhibition of combination	CI value	Dose (mM) Acarbose	Dose (mM) Kaempferol	DRI Acarbose	DRI Kaempferol
26	0.795 (syn)	0.027	1.99	2.39	2.66
53.7	0.900 (syn)	0.068	5.49	2.12	2.33
71.1	0.972 (syn)	0.119	10.4	1.97	2.15
86	0.922 (syn)	0.239	22.6	2.07	2.27
91	1.82 (ant)	0.348	34.5	1.09	1.11

^a^ Fa is the fraction affected. CI lower than, equal to, or greater than 1 signifies synergism (syn), addition (add), or antagonism (ant), respectively. DRI > 1 is favored and indicative of fold-change in dose reduction for the drug in the combination

Similarly, acarbose amalgamated with kaempferol also demonstrated remarkable synergism with a CI < 1 (Table 4) and (Fig. 4C) at all effect levels sans the 90% level. Likewise, in the dose normalized isobologram, the 90% combination data point was located above the additive line (Fig. 4D), stipulating antagonistic behavior at high doses. Due to the synergy present between acarbose and kaempferol at lower doses, an average of a two-fold dose reduction was observed (Table 4). The most prominent reduction was noted at the 10% effect level, as the concentration needed was 2.39- and 2.66-fold lower in magnitude, respectively, to produce the required inhibitory activity.

### Molecular interactions of kaempferol and acarbose

In this section, kaempferol; the top chosen in vitro hit and acarbose; the synthetic inhibitor both were docked with human pancreatic α-amylase to support in vitro studies and predict protein–ligand interactions. As shown in Table 5, acarbose gave -7.5 kcal/mol and kaempferol, -8.1 kcal/mol interaction energy indicating stable binding. As shown in Figure S8A, B, acarbose key residues formed hydrogen bonds with GLN63, ASP197, GLU233, and ASP300. Kaempferol, on the other hand, was engaged by two hydrogen bonds with GLN63 and a π-π T-shaped interaction with TRP59, as shown in Figure S8C, D, resembling that formed with the co-crystallized ligand myricetin found in the 4GQR structure (Figure S9). In addition, kaempferol formed an extra hydrogen bond with ASP300 and a π-π stacking interaction with TYR62. Details of other interactions of acarbose and kaempferol with α-amylase are depicted in Table 5.

**Table 5:** Binding energies and interaction details of acarbose and kaempferol with human pancreatic α-amylase (PDB ID: 4GQR), individually and in combination of each other

Ligands		Binding energy (kcal/mol)	Contact residues	
			H bonds (bond length)	Hydrophobic
Individually	Acarbose	-7.5	GLN63 (5.04 Å), THR163 (3.58 Å), ASP197 (3.38 Å), GLU233 (4.90 Å), HIS299 (4.92 Å), ASP300 (3.78 Å)	ILE51, ASN53, PRO54, TRP58, TRP59, TYR62, VAL98, HIS101, GLY104, ALA106, VAL107, LEU162, ARG195, ALA198, PHE256, HIS305
	Kaempferol	-8.1	GLN63 (4.32 Å) and ASP300 (5.18 Å)	TRP58, TRP59, TYR62, HIS101, LEU162, LEU165, ARG195, ALA198, GLU233, ILE235, HIS299
In Combination	Acarbose (co-ligand)	-7.1	GLU149 (5.55 Å), TYR151 (4.87 and 4.44 Å), THR163 (3.93 Å), LYS200 (6.01 Å), HIS201 (5.11 Å), GLU240 (3.93 and 4.90 Å), ASP300 (3.81 and 3.85 Å), HIS305 (3.59 and 4.16 Å)	TRP58, ASN150, ASN152, LEU162, ILE235, GLY360, ALA307, Kaempferol
	Kaempferol (co-ligand)	-9.3	GLN63 (4.63 and 6.01 Å), ASP197 (4.07 Å)	TRP58, TRP59, TYR62, HIS101, LEU162, LEU165, ARG195, ALA198, GLU233, ILE235, HIS299, Acarbose

#### Cooperative binding of acarbose and kaempferol using molecular docking

**Fig. 5:**
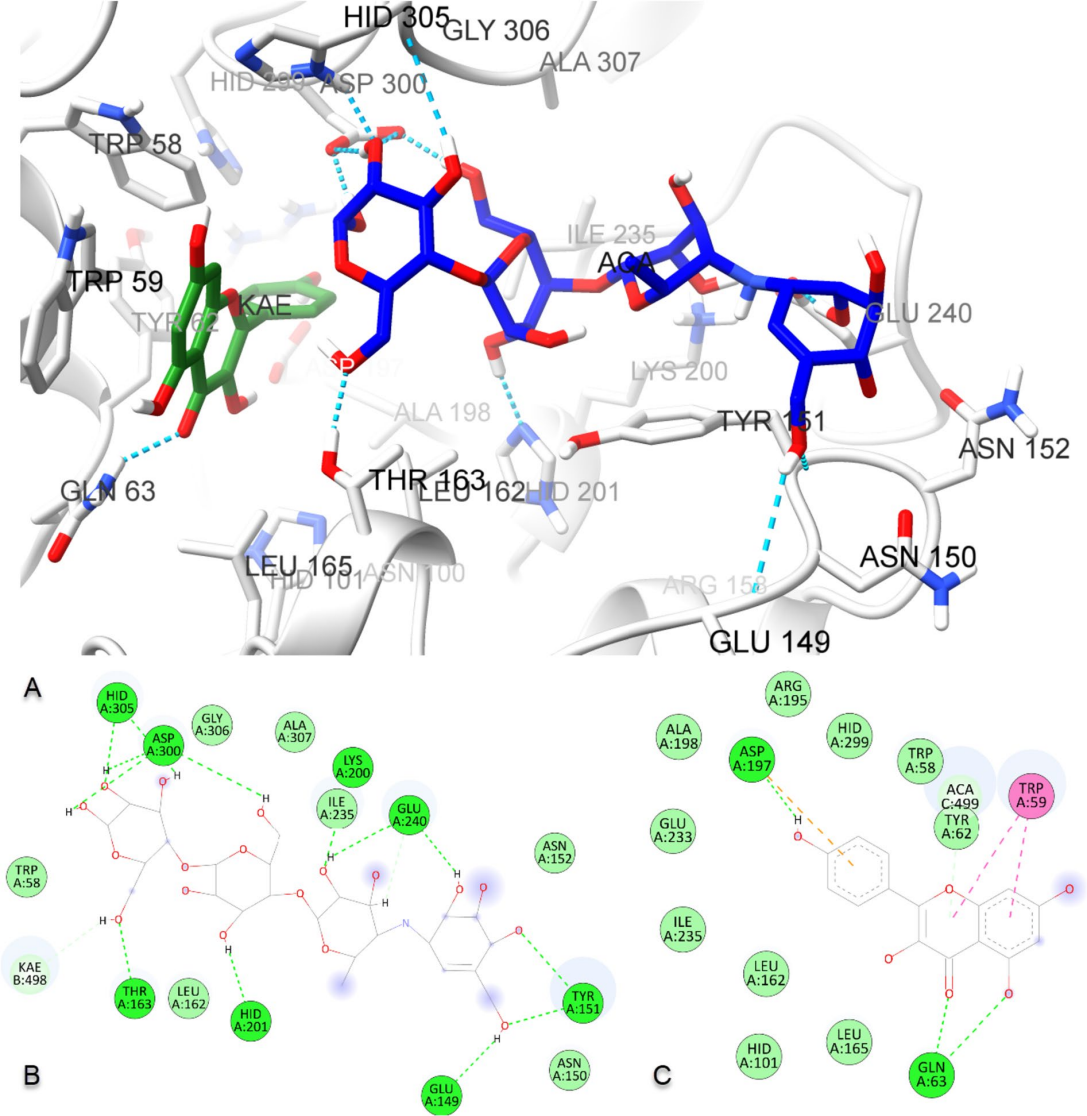
The binding mode of kaempferol and acarbose complex at the active site of human pancreatic α-amylase (PDB ID: 4GQR) obtained with AutoDock Vina (**A)**, and schematic protein–ligand interaction diagrams of acarbose (**B**) and kaempferol (**C**), respectively

The synergistic effect of acarbose and kaempferol as the most active inhibitor on α-amylase was modeled as revealed from previous in vitro assay results. To perform molecular docking of the combination, acarbose was docked to the protein–ligand complex of α-amylase and kaempferol (Fig. 5). Then, kaempferol was removed from this triple structure and redocked again. As shown in Fig. 5A, B, acarbose shows hydrogen bond interaction with ASP300 and THR163, and carbon-hydrogen bond interaction with kaempferol, enhancing the assumption of cooperative binding in the active site. Kaempferol, on the other hand, established van der Waals interactions with the co-ligand acarbose, two hydrogen bonds with GLN63, one hydrogen bond with ASP197, and π-π interactions with TRP59, as shown in Fig. 5A-C. While kaempferol alone generated an interaction energy of -8.1 kcal/mol, when combined with acarbose, the energy was minimized to -9.3 kcal/mol, indicating that the binding power of kaempferol to α-amylase was further augmented (Table 5).

### Molecular dynamics simulations

**Fig. 6:**
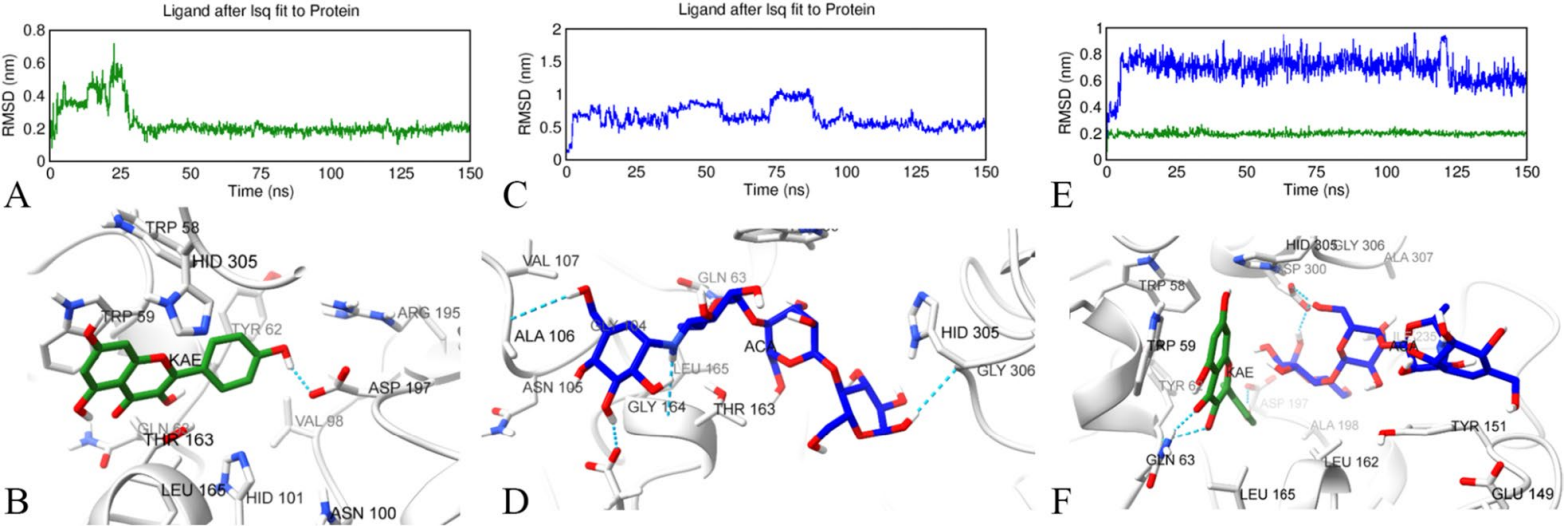
Molecular dynamics simulations’ trajectory analysis. The root mean square deviation (RMSD) plot showing conformational changes of kaempferol (**A**), acarbose (**C**), and the co-ligand acarbose and kaempferol (**E**) at the active site of human pancreatic α-amylase, respectively. Binding poses of kaempferol (**B**), acarbose (**D**), and the co-ligand kaempferol and acarbose together (**F**) at the active pocket of α-amylase at the 150^th^ ns

Molecular dynamics (MD) simulations were performed to deeply understand and examine the stability of α-amylase with acarbose, kaempferol, and the combination of acarbose and kaempferol protein–ligand complexes obtained from molecular docking studies [86]. MD simulations allow us to visualize the physical interactions occurring in the docked complexes and provides us with the ability to observe the conformational and structural changes transpiring in the protein and the ligand throughout the duration of the simulation. Root mean square deviation (RMSD) is a frequently used method to numerically explain the changes in the protein active pocket of ligands in protein–ligand complexes. In this study, conformational changes and mobility, namely stability, of acarbose, kaempferol, and acarbose – kaempferol combination were investigated according to α-amylase active site residues during the 150 ns simulation. First, individual drug complexes with the enzyme were analyzed. As shown in Fig. 6A, kaempferol stabilized after the first 30 ns and remained stable at 0.2 nm with a mean RMSD value of 0.23 ± 0.09 nm. The protein–ligand binding poses at the end of the MD simulation were analyzed to examine the interaction changes. As given in Fig. 6B, the hydrogen bond of kaempferol with GLN63 did not persist, while the hydrogen bond with ASP197 remained stable. An animated video for the MD trajectory to visualize protein–ligand interactions between 0 and 150 ns was created. In this way, their interaction every 0.5 ns for 150 ns was visualized in the Electronic supplementary information. Kaempferol remained stable after the first 30 ns, as shown in Video S1 confirming the previous findings. Secondly, the stability of acarbose with α-amylase and the protein–ligand interaction changes were analyzed. As given in Fig. 6C, RMSD of its complex was found to be below 0.9 nm with a mean value of 0.80 ± 0.22 nm. The binding mode of acarbose at 150 ns is given in Fig. 6D. Compared to the molecular docking pose, it was observed that the hydrogen bonds with ASP300, ASP197, and GLU233 were broken; however, acarbose remained in the active pocket by forming new hydrogen bonds and interactions as shown in Video S2.

Finally, the stability and behavior of the combination of acarbose and kaempferol with α-amylase were analyzed by MD simulation. According to the RMSD data given in Fig. 6E, kaempferol was clearly stable at 0.2 nm and the mean RMSD value was 0.19 ± 0.01 nm, while acarbose was below 0.8 nm after the first 10 nm and the mean RMSD value was 0.69 ± 0.10 nm. Hence, the RMSD values of the combination were surprisingly low compared to the individual drugs indicating elevated stability. The interactions of co-ligand acarbose and kaempferol MD with α-amylase at the end of the simulation are given in Fig. 6F. Interestingly, it was understood that the protein–ligand molecular docking interactions given in Fig. 5 were completely preserved for kaempferol, especially the hydrogen bonds formed with GLN63 and ASP197 and remained potently stable, while acarbose also preserved the hydrogen bond with ASP300. It can be concluded that acarbose and kaempferol combination with α-amylase complex stabilized each other by forming synergism as given in Video S3.

#### MM-PBSA Binding free energy calculations

One of the important ways to numerically describe protein–ligand stability in MD simulations is to measure the binding-free energy MM-PBSA [87]. In this study, MM-PBSA measurement of acarbose, kaempferol, and acarbose–kaempferol combination with human pancreatic α-amylase was calculated from 1500 frames between 0 and 150 ns. As given in Table 6, the protein–ligand complexes of α-amylase and acarbose, α-amylase and kaempferol, and α-amylase and co-ligand acarbose and kaempferol MM-PBSA values were -19.06 ± 7.11 kcal/mol, -25.90 ± 3.68 kcal/mol, and -52.63 ± 7.02 kcal/mol, respectively. The sum of MM-PBSA values of α-amylase and acarbose, and α-amylase and kaempferol complexes (-44.96 kcal/mol) was higher than that of α-amylase complex with co-ligand acarbose and kaempferol (-52.63 kcal/mol) which means that acarbose and kaempferol combination produced more interactions.

**Table 6:** Binding free energy MM-PBSA computations of α-amylase with kaempferol, acarbose, and combination of kaempferol and acarbose from 1500 frames between 0 and 150 ns

Energy component	Average energy (kcal/mol)		
	α-amylase & acarbose	α-amylase & kaempferol	α-amylase & kaempferol and acarbose
ΔVDWAALS	-32.89 ± 5.03	-26.35 ± 3.01	-57.97 ± 4.78
ΔEEL	-31.56 ± 14.40	-37.79 ± 8.14	-73.64 ± 13.85
ΔEGB	50.46 ± 11.04	42.11 ± 4.71	88.09 ± 9.09
ΔESURF	-5.07 ± 0.74	-3.87 ± 0.26	-9.12 ± 0.46
ΔGGAS	-64.45 ± 15.60	-64.14 ± 7.61	-131.60 ± 14.05
ΔGSOLV	45.39 ± 10.58	38.24 ± 4.61	78.98 ± 8.83
ΔTOTAL	-19.06 ± 7.11	-25.90 ± 3.68	-52.63 ± 7.02

Δ: Complex—Receptor—Ligand, *VDWAALS* van der Waals, *EEL* electrostatic Energy, *EGB* electrostatic solvation free energy evaluated from the generalized Born equation, *ESURF* the nonpolar component of the solvation energy, *GGAS* gas-phase energy, *GSOLV* solvation free energy

To sum up, considering the MD trajectory analyses, RMSD, time-dependent changes in binding modes, and MM-PBSA measurements, it was concluded that the combination of acarbose and kaempferol gave more potent protein–ligand interactions than acarbose or kaempferol alone.

## Conclusion

In this study, GC–MS analysis uncovered the presence of 15 compounds that were previously unreported in Egyptian propolis, including aucubin which has never been reported in propolis worldwide. In another first of its kind, the anti- α-amylase activity of Egyptian propolis was investigated through in silico docking. Additionally, this in silico study included an unprecedented investigation on the inhibitory α-amylase potential of aucubin, which showed promising results. To further reiterate these findings, in vitro assay followed and the antidiabetic potential of aucubin was verified for the first time via an in vitro assay. Kaempferol also showed positive outcomes with its being the second most potent α-amylase inhibitor. Thereafter, combination therapy was conducted using kaempferol due to the fragile and unstable nature of aucubin which hinders its possible utilization in lab or in pharmaceutical preparations. Through combination therapy analysis, synergistic behaviour was found in both the binary combination of acarbose with propolis and with kaempferol, especially at lower doses. Molecular dynamics simulations were executed to examine the stability of α-amylase with the combination of acarbose and kaempferol protein–ligand complexes. The stability of this protein–ligand complex was confirmed through the interactions manifesting between the acarbose and kaempferol combination, thus indicating synergistic behavior. These findings suggest a potential multidrug regimen that requires less than half the dose of a conventional drug to achieve the same inhibitory level. Through use of this multidrug regimen, and due to the decreased dose of acarbose used, it is postulated that the side effects of acarbose would in turn decrease, improving patient adherance and subsequently, therapeutic outcomes. Further pharmacological in vivo investigations in alloxan- or streptozotocin-induced diabetic mice or rats are required to confirm the potent antidiabetic potential of the studied combination, before ultimately investigating the regimen through clinical trials.

### Supplementary Information

**Additional file 1.**

**Additional file 2.**

## Data Availability

All data generated or analyzed during this study are included in this article (and its supplementary information files).

## Abbreviations

DM: Diabetes Mellitus
MD: Molecular Dynamics
AACE: American Association of Clinical Endocrinology
IR: Infrared
NMR: Nuclear Magnetic Resonance
UV: Ultraviolet
HPLC: High‐Performance Liquid Chromatography
HPTLC: High‐Performance Thin Layer Chromatography
GC: Gas Chromatography
GC–MS: Gas Chromatography coupled with Mass Spectrometry
DNS: 3,5-Dinitrosalicylic Acid
BSTFA: N,O-Bis(Trimethylsilyl)Trifluoroacetamide
EI: Electron Ionization
PDB: Protein Data Bank
4GQR: Human Pancreatic α-amylase complexed with Myricetin
3OLD: Human Pancreatic α-amylase complexed with Acarviostatin I03
1OSE: Porcine Pancreatic α-amylase complexed with Acarbose
XP mode: Extra-Precision
CI: Combination Index
DRI: Dose Reduction Index
RMSD: Root Mean Square Deviation
MM-PBSA: Molecular Mechanics Poisson-Boltzmann Surface Area
TMS: Trimethylsilyl
