# Supplementary material for: Synergistic effect of potential alpha-amylase inhibitors from Egyptian propolis with acarbose using in silico and in vitro combination analysis
Source: BMC Complement Med Ther. 2024 Jan 30;24:65. doi: 10.1186/s12906-024-04348-x (PMC10826043; doi:10.1186/s12906-024-04348-x)
Supplement: Supplementary file 1 — Additional file 1. [file 12906_2024_4348_MOESM1_ESM.zip › 01-06-24 Supplementary Materials BMC.docx]

**Synergistic effect of potential alpha-amylase inhibitors from Egyptian propolis with acarbose using *in silico* and *in vitro* combination analysis**

**Supplementary materials**

**Results and discussion**

**EI-MS analysis of the major compounds in derivatized propolis extract**

**Fragmentation pattern of d-fructose**

D-fructose (13.77%) was the most abundant constituent in the Egyptian propolis sample, it was the fourth constituent to be eluted at a retention time of 4.36 minutes. Its molecular ion peak is *m/z* 540 and its mass spectrum can be seen at Figure S2A.

In accordance with the derivatization of the propolis sample by silylation, numerous derivative ion peaks are found throughout the spectra. In the case of d-fructose, a handful of ion peaks can be characterized at *m/z* 73, [TMS] ^+^; *m/z* 204, resulting from Mclafferty Rearrangement of TMS group as shown in Figure S3 [1]; and *m/z* 217, reflecting the degradation of furanose ring shown in Figure S4 [1].

**Fragmentation pattern of d-glucopyranose and hexopyranose**

D-glucopyranose (10.59%) and hexopyranose (10.08%) were eluted at 5.66 minutes and 6.5 minutes, respectively. They both shared a molecular ion peak of m/z 540 and their mass spectra can be seen at Figure S2B and Figure S2C, respectively.

D-glucopyranose and hexopyranose exhibit similar ion peaks that are identified at *m/z* 73, [TMS] ^+^; *m/z* 191, *m/z* 204 and *m/z* 307, revealing the fragments resulting from degradation of pyranose ring as shown in Figure S5 [1]. Additionally, d-glucopyranose displays ion peaks *m/z* 169, [M-4TMSO-CH_3_] ^+^; and *m/z* 361, [M-TMSO-6CH_3_] ^+^.

**Fragmentation pattern of chrysin**

The most abundant flavonoid present in the sample was chrysin which was eluted at 18.04 minutes. It has a molecular ion peak of 398. Its mass spectrum is displayed in Figure S2D.

Chrysin fragmentation pattern is manifested as ion peaks *m/z* 311, [M+TMS–CH4]; *m/z* 327, [M+TMS]^+^; *m/z* 383, [M+2TMS–CH4]^+^; *m/z* 399, [M+2TMS–H]^+^; and *m/z* 471, [M+3TMS–H]^+^ as illustrated in Figure S6 [2].


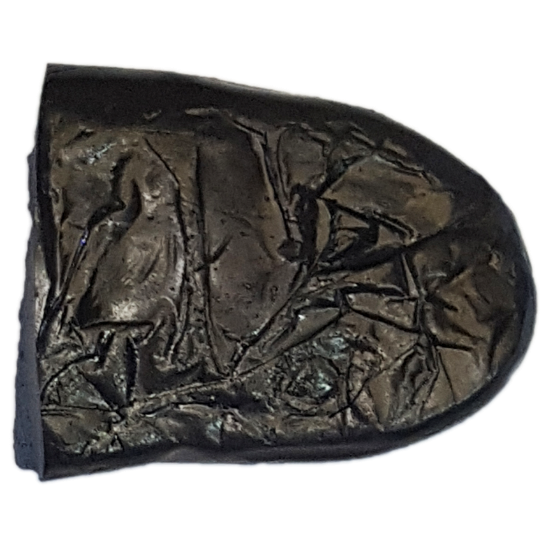


**Figure S1** Egyptian propolis sample under investigation obtained from the apiary mentioned in the experimental section.


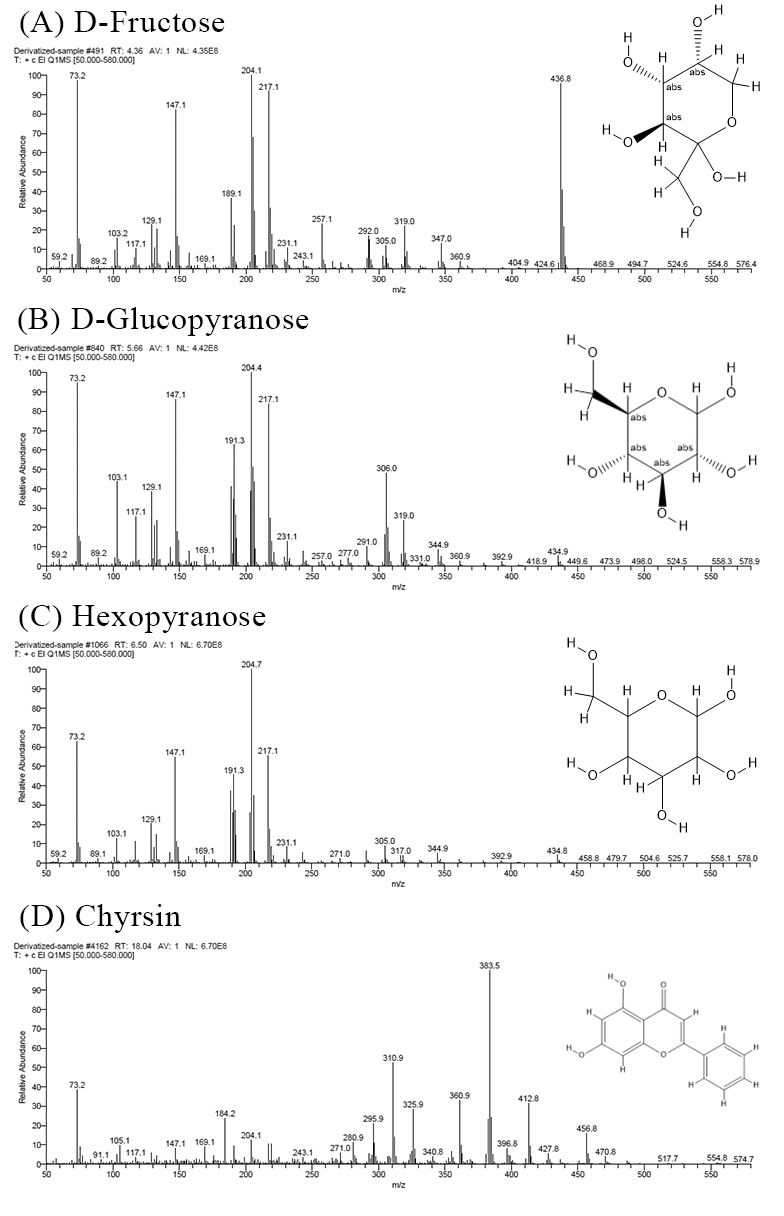


**Figure S2** EI-Mass spectra of d-fructose **(A)**, d-glucopyranose **(B)**, hexopyranose **(C)**, and chrysin **(D)**.


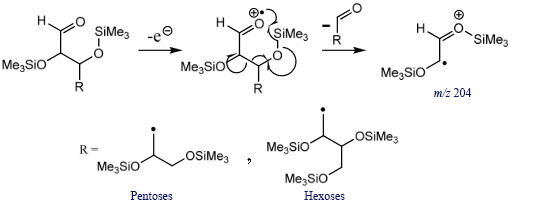


Figure S3 Mclafferty Rearrangement of TMS in sugar molecule.

Adapted from Mogoşanu G, Grumezescu A, Mihaiescu D, Istrati D, Mogosanu D, Buteicǎ S. Identification of sugars from Silene Albae Herba using GC-MS technique. UPB Sci Bull Ser B Chem Mater Sci. 2011;73:101–8.


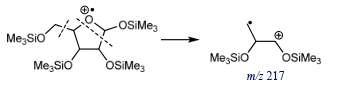


Figure S4 Degradation of silylated furanose sugar molecule.

Adapted from Mogoşanu G, Grumezescu A, Mihaiescu D, Istrati D, Mogosanu D, Buteicǎ S. Identification of sugars from Silene Albae Herba using GC-MS technique. UPB Sci Bull Ser B Chem Mater Sci. 2011;73:101–8.


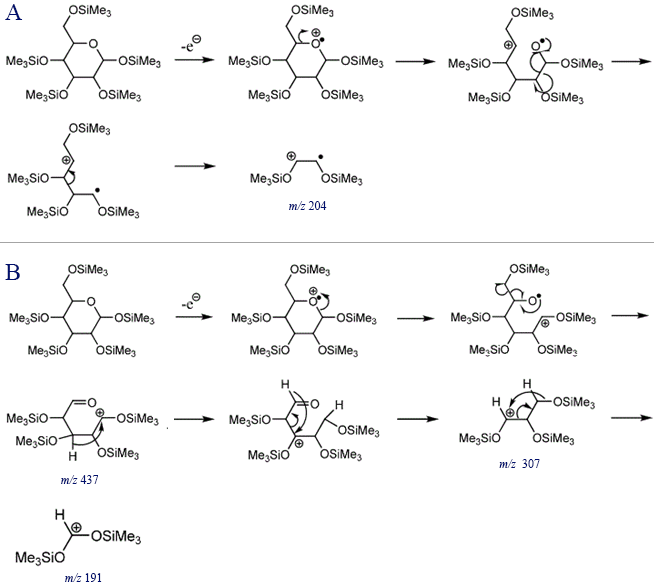


Figure S5 Degradation of silylated pyranose sugar molecule.

Adapted from Mogoşanu G, Grumezescu A, Mihaiescu D, Istrati D, Mogosanu D, Buteicǎ S. Identification of sugars from Silene Albae Herba using GC-MS technique. UPB Sci Bull Ser B Chem Mater Sci. 2011;73:101–8.


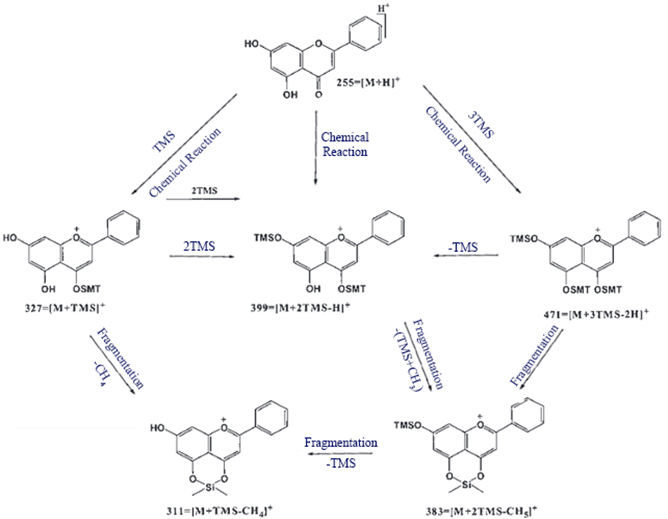


Figure S6 Chrysin fragmentation pattern.

Adapted from Kim SJ, Kumar AP, Lee YI. Enhanced detection and structural characterization of flavonoids by complexation with N,O-bis(trimethysilyl)trifluoroacetamide using electrospray ionization mass spectrometry. Anal Sci. 2008;24:1177–82.

**
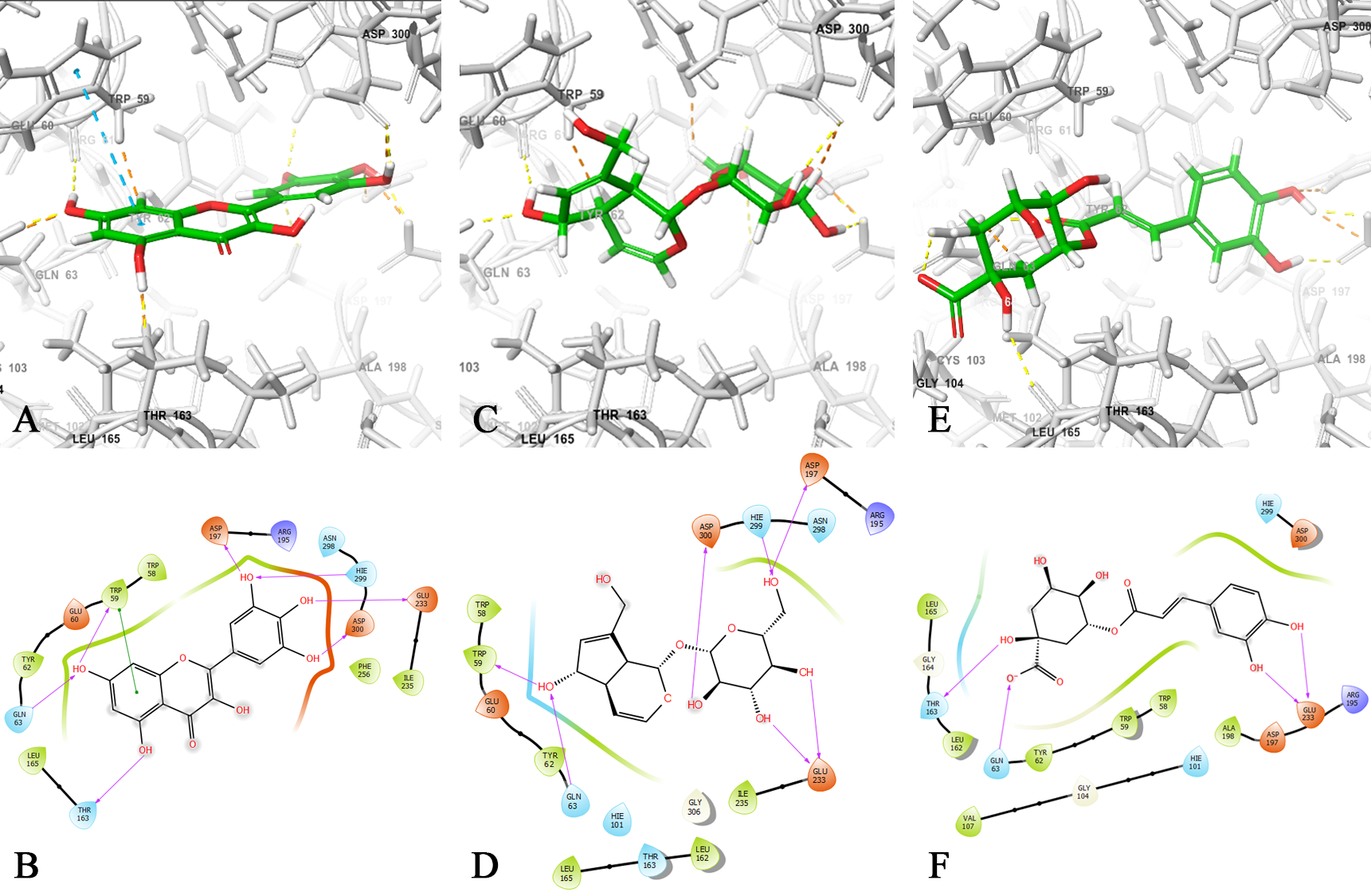
**

Figure S7 Molecular docking poses and protein-ligand interaction diagrams of α-amylase (PDB ID: 4GQR) with myricetin (A-B), aucubin (C-D), and chlorogenic acid (E-F).


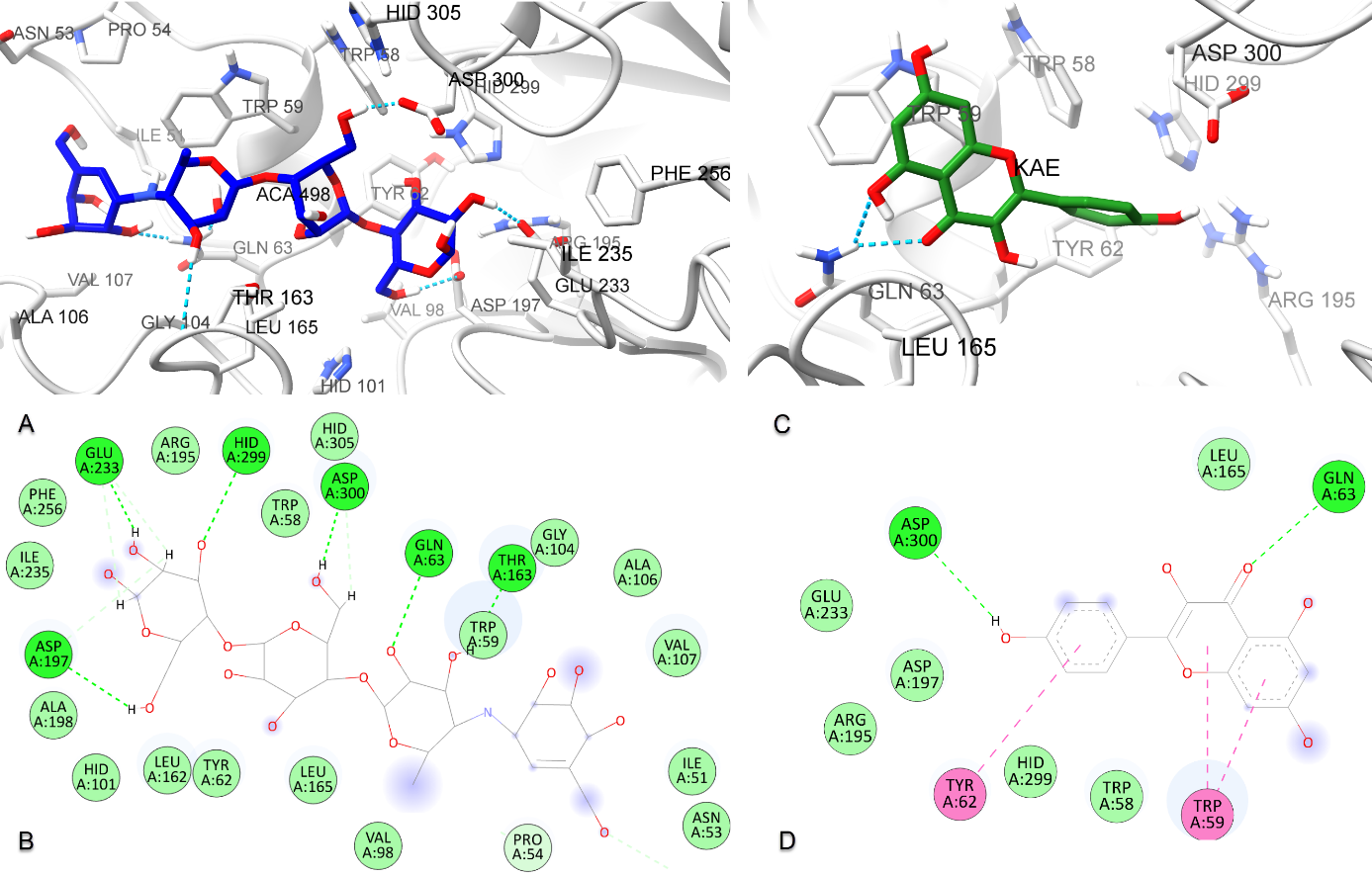


**Figure S8** Molecular docking poses and protein-ligand interaction diagrams of α-amylase (PDB ID: 4GQR) with standard compound acarbose **(A-B)**, and kaempferol **(C-D)**.

**
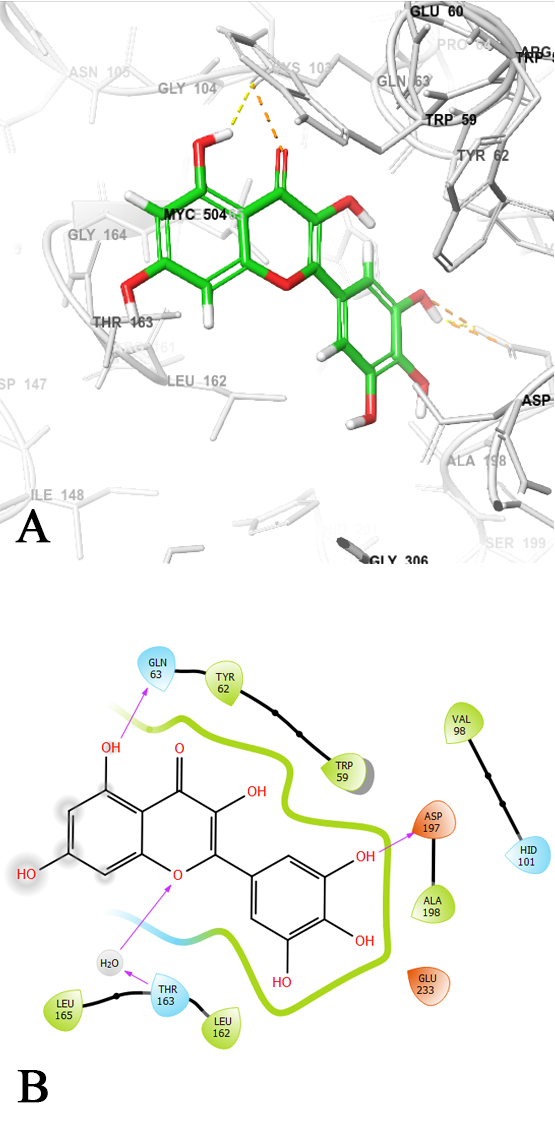
**

Figure S9 Molecular docking poses and protein-ligand interaction diagrams of α-amylase (PDB ID: 4GQR) with cocrystallized ligand, myricetin (A-B).

**Sheet S1** In-house library of compounds naturally occurring in Egyptian propolis.

Sheet S1.xlsx

**Table S1** Structures and IC_50_ of the 40 compounds constituting the validation set of the docking process.

| # | Compound Name | IC_50_ | Structure | Reference |
| --- | --- | --- | --- | --- |
| 1 | Acarbose | 1.49 ± 0.03 μM | 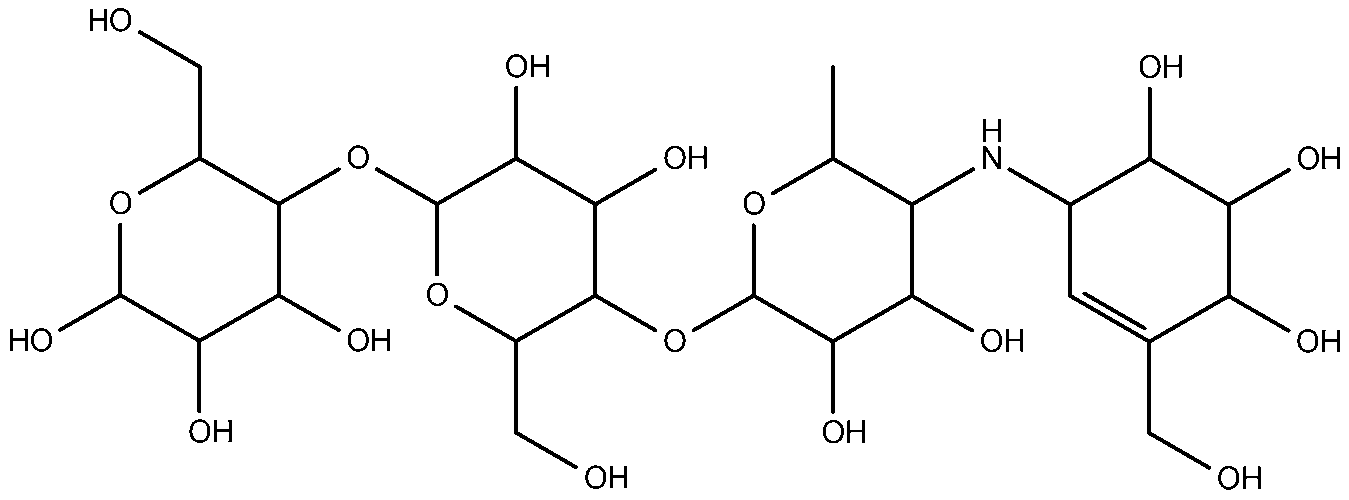 | [3] |
| 2 | Quercetagetin-7-O-β-D-glucopyranoside | 8.04 ± 0.69 μM | 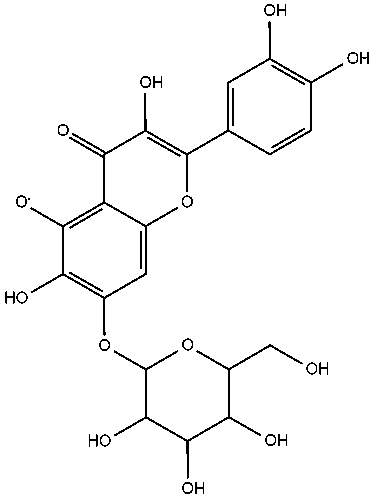 | [3] |
| 3 | Apigenin-7-O-β-D-glucopyranuronide | 10.41 ± 2.51 μM | 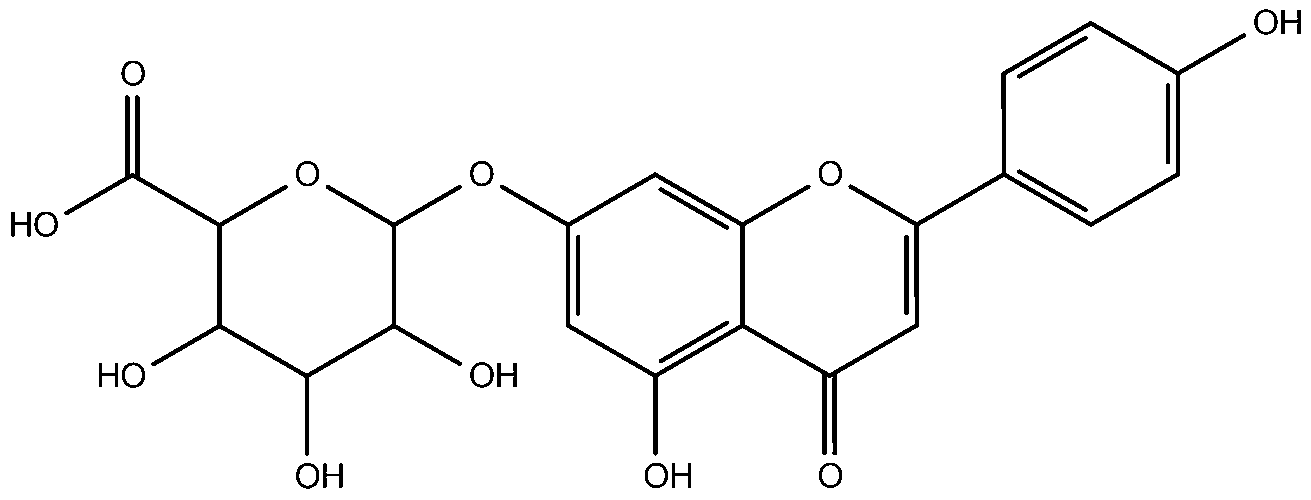 | [3] |
| 4 | Epigallocatechin gallate | 11.5 ± 0.02 μM | 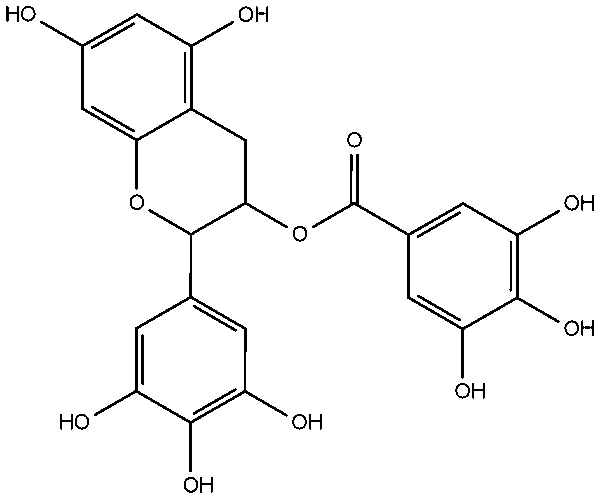 | [4] |
| 5 | Apigenin | 12.15 ± 2.14 μM | 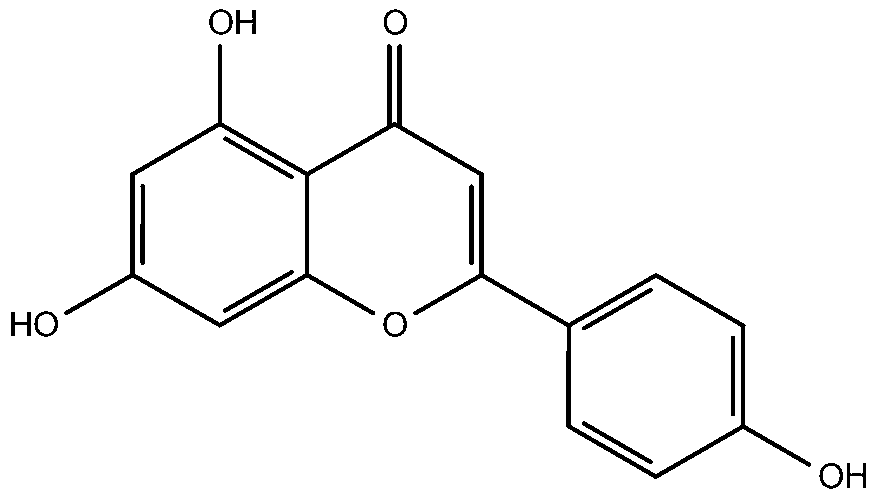 | [3] |
| 6 | Quercetin-3-monomethyl ether | 13.59 ± 2.04 μM | 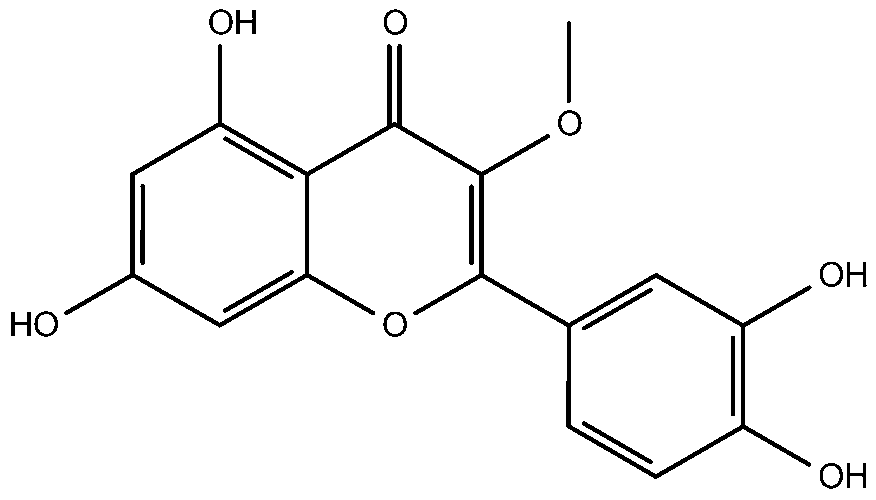 | [3] |
| 7 | Quercetin | 14.11 ± 1.96 μM | 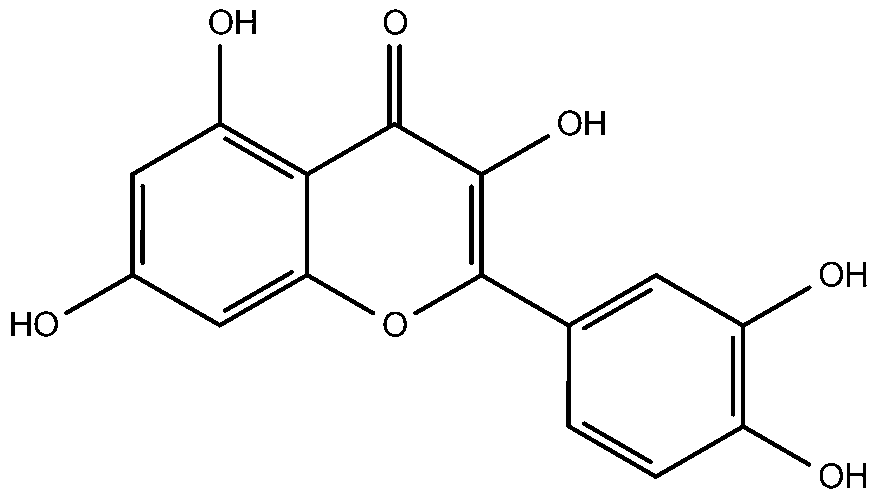 | [3] |
| 8 | Luteolin | 14.57 ± 0.74 μM | 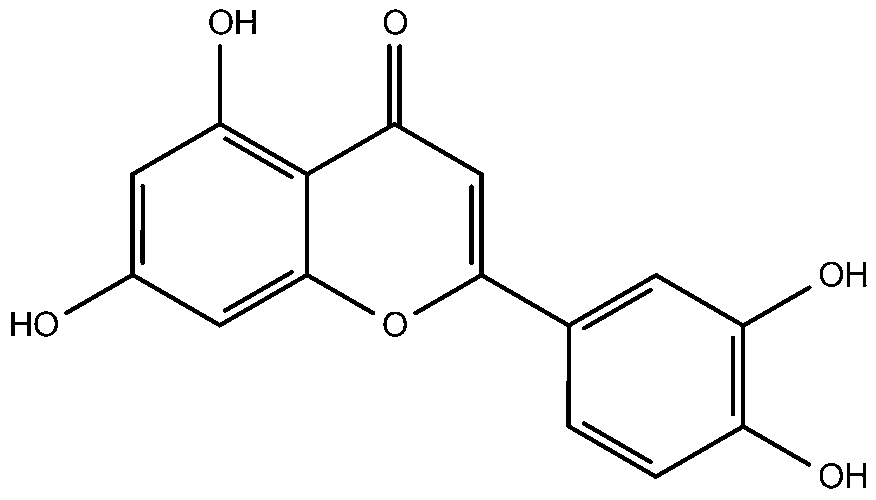 | [3] |
| 9 | 7, 3', 4'-trihydroxyflavone | 16.31 ± 0.52 μM | 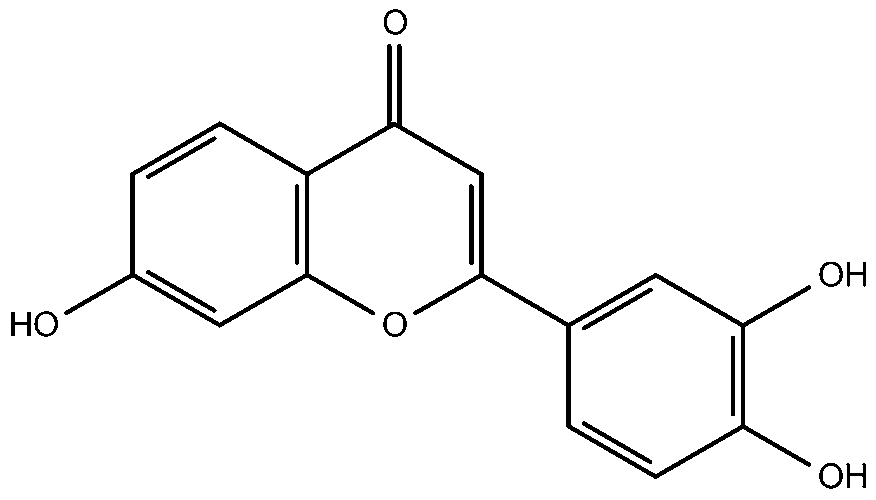 | [3] |
| 10 | Kaempferol | 16.48 ± 1.84 μM | 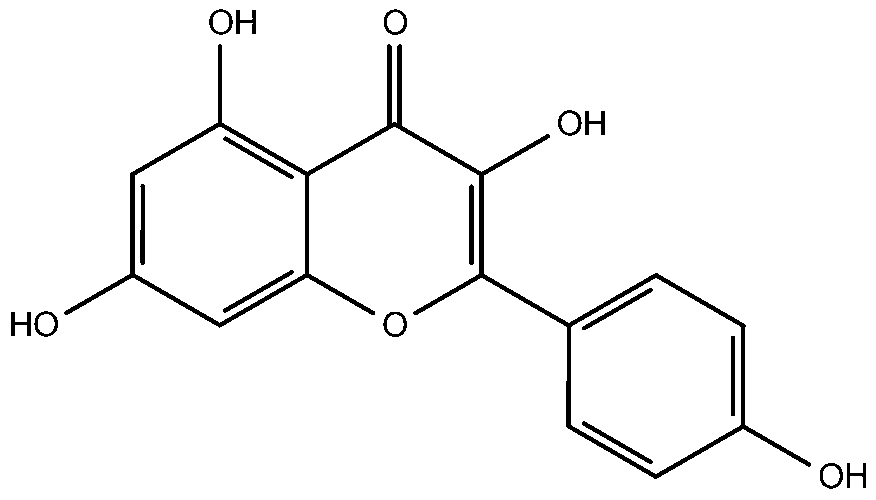 | [3] |
| 11 | Naringenin | 21.85 ± 3.21 μM | 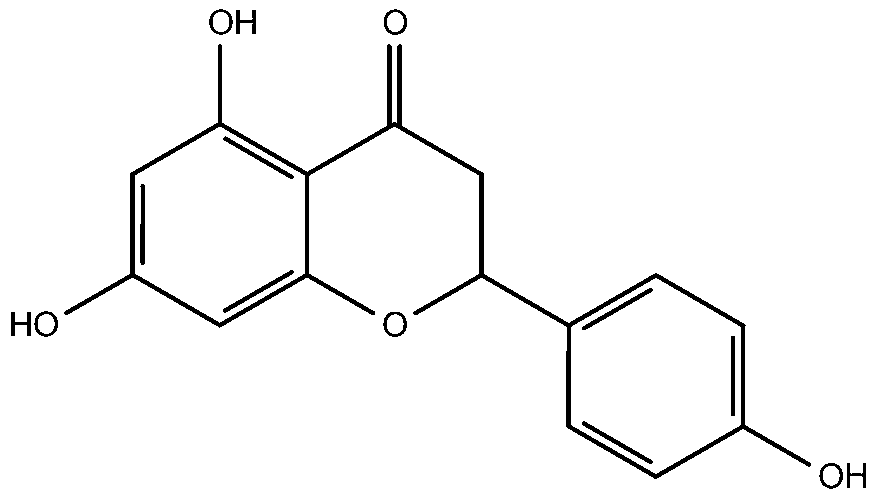 | [3] |
| 12 | Kaempferide | 22.9 ± 3.17 μM | 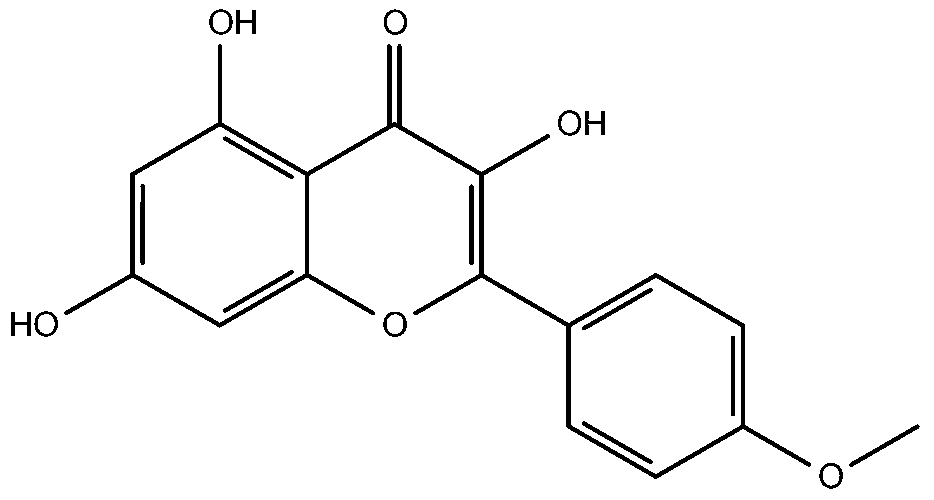 | [3] |
| 13 | Cyanidin-3-glucoside | 24 ± 3 μM | 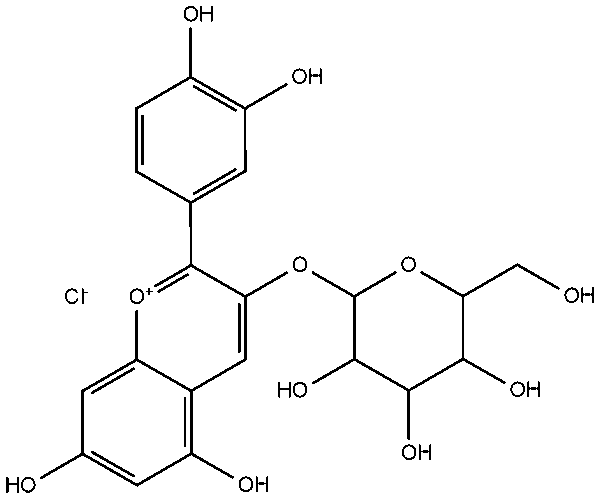 | [5] |
| 14 | Cyanidin-3-rutinoside | 24.4 ± 0.1 μM | 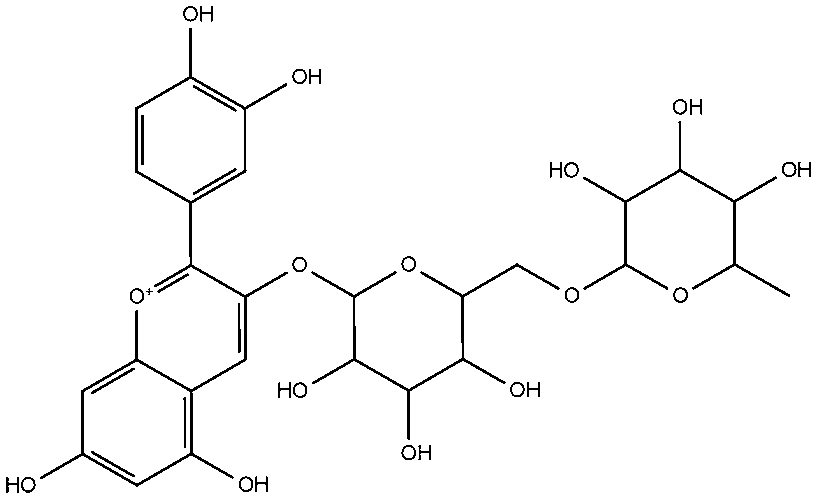 | [6] |
| 15 | Gallic acid | 27.79 ± 2.06 µM | 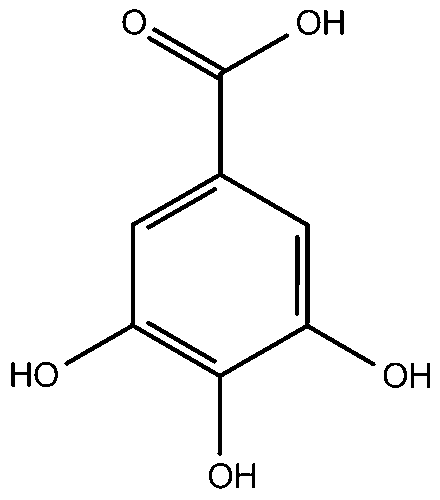 | [7] |
| 16 | L-epicatechin | 31.46 ± 2.21 μM | 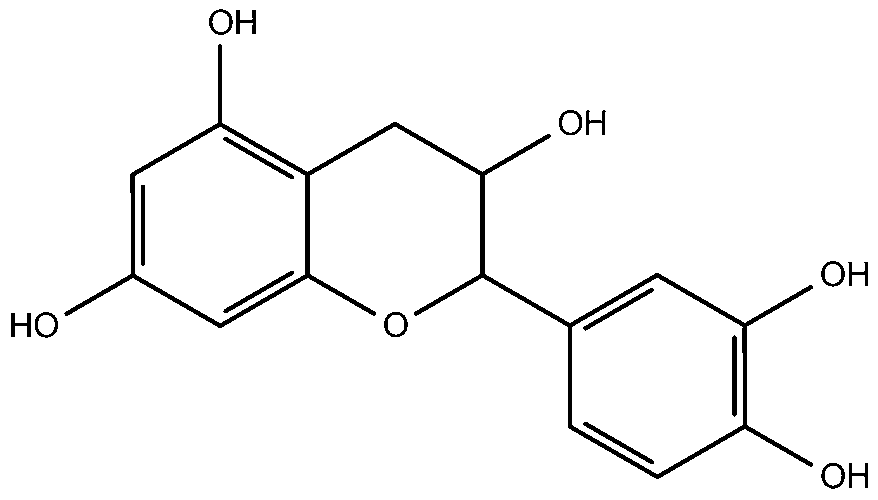 | [3] |
| 17 | Cyanidin-3,5-glucoside | 40 ± 7 μM | 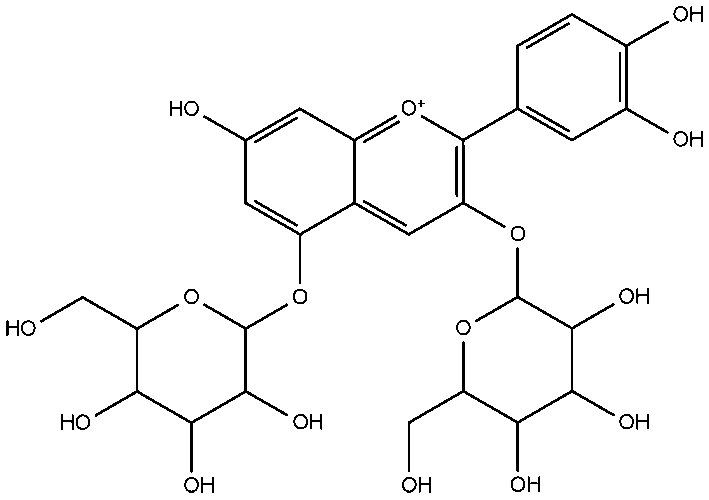 | [5] |
| 18 | 6-Chloro-3',4',5,7-tetrahydroxyflavone | 44 ± 3 μM | 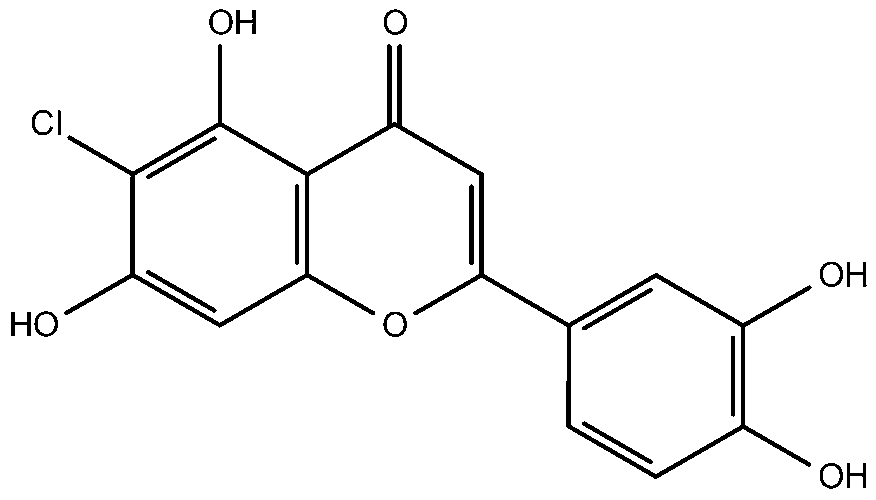 | [8] |
| 19 | 3,7,4'-Trihydroxyflavone | 59 ± 4 μM | 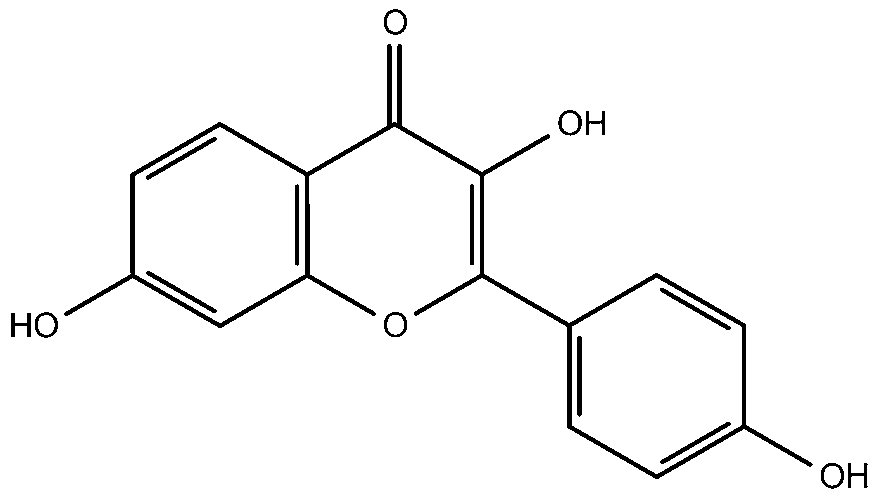 | [8] |
| 20 | Peonidin-3-glucoside | 75 ± 7 μM | 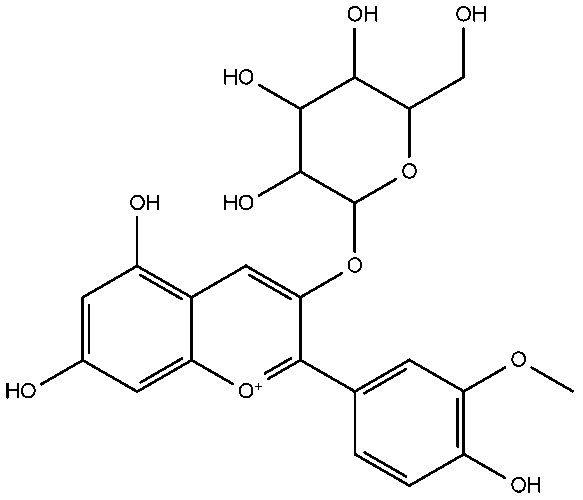 | [5] |
| 21 | Myricetin | 107 ± 6 μM | 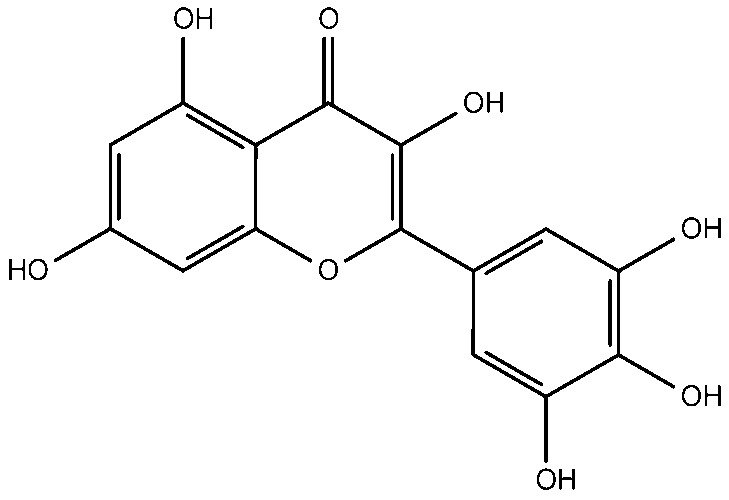 | [8] |
| 22 | 5,3',4'-Trihydroxyflavone | 148 ± 5 μM | 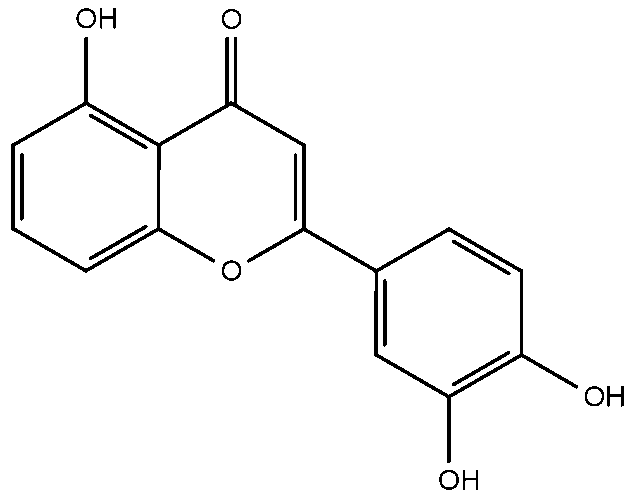 | [8] |
| 23 | Chrysoeriol | 192 ± 7 μM | 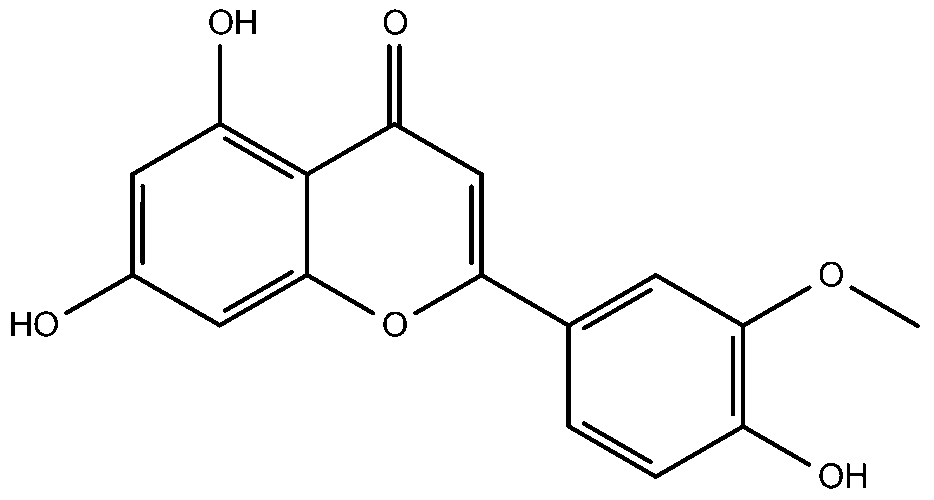 | [8] |
| 24 | Chlorogenic acid | 1051 ± 18 μM | 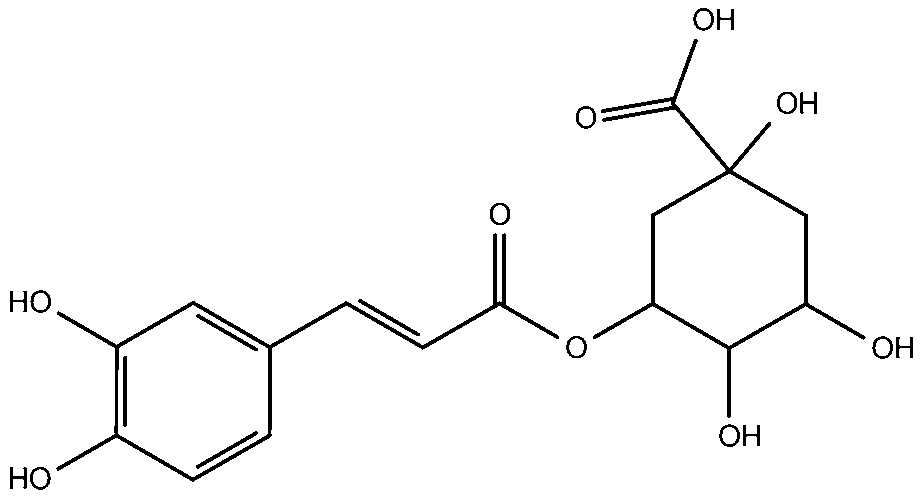 | [9] |
| 25 | Catechin | 1156 ± 24 μM | 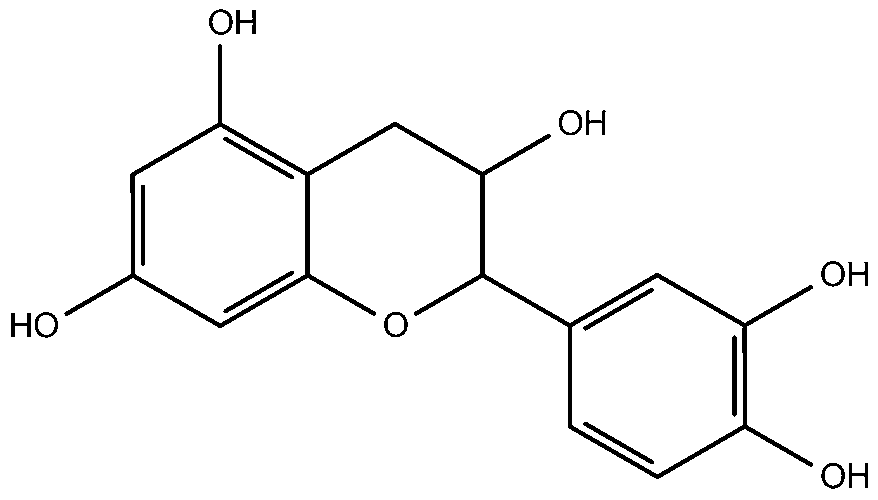 | [9] |
| 26 | Syringic acid | 1980 ± 10 μM | 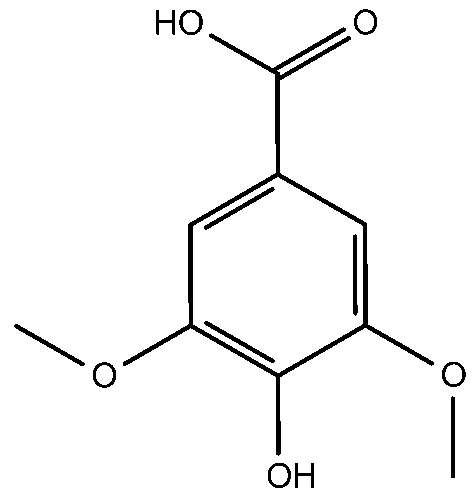 | [10] |
| 27 | Vanillic acid | 2050 ± 10 μM | 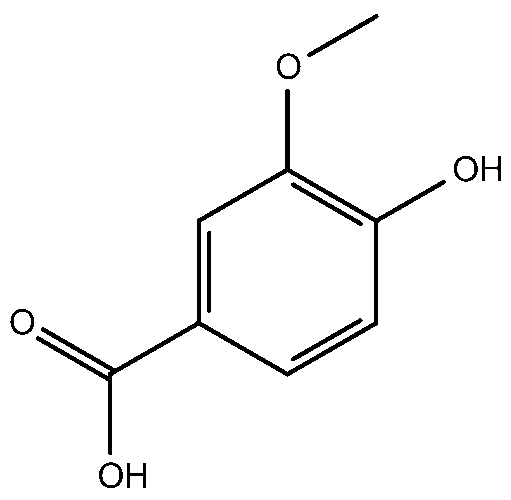 | [10] |
| 28 | 5,7-dihydroxy-3-(4′-hydroxybenzyl) chromone | 2590 ± 50 μM | 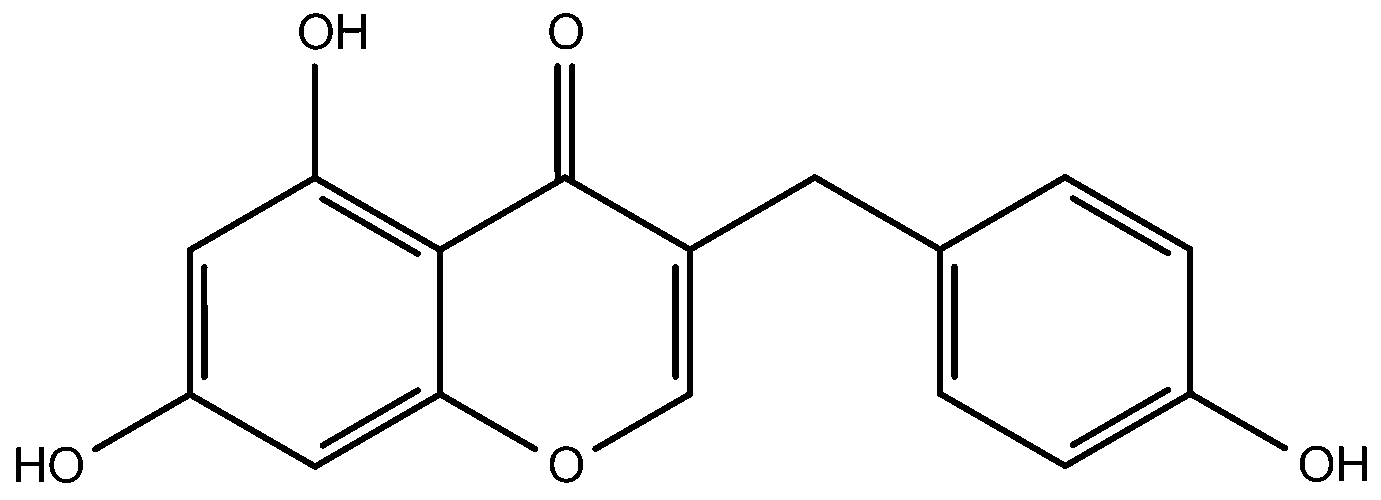 | [11] |
| 29 | Protocatechuic acid | 2650 ± 160 μM | 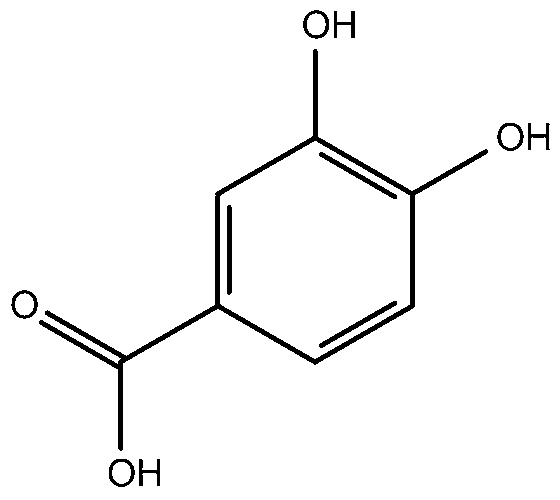 | [10] |
| 30 | Diosmetin | 3797 ± 486 μM | 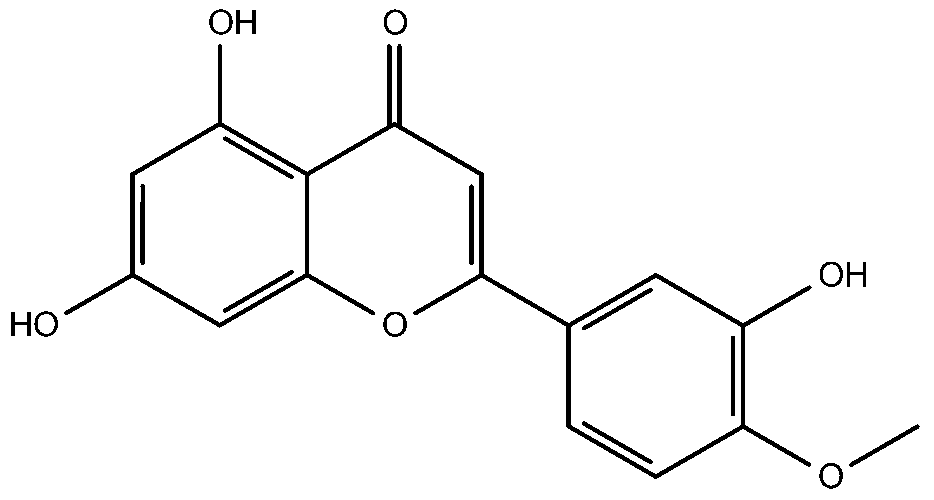 | [12] |
| 31 | 5,7-dihydroxy-6-methyl-3-(4′-hydroxybenzyl) chroman-4-one | 5990 ± 60 μM | 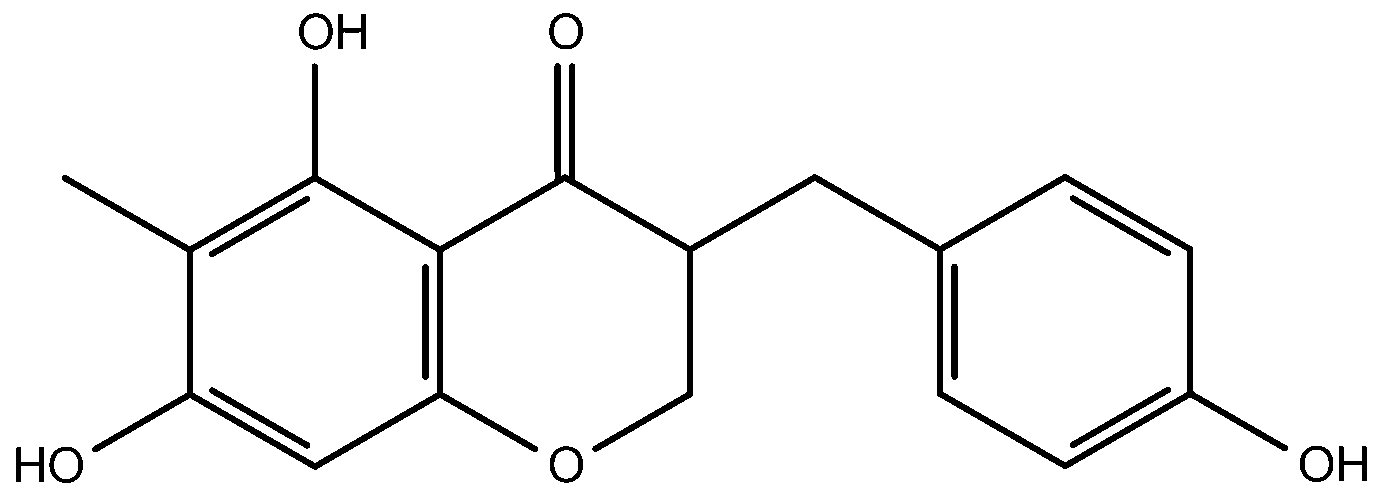 | [11] |
| 32 | (2S)-4′-hydroxy-5,7-dimethoxy-8-methylflavan | 6030 ± 270 μM | 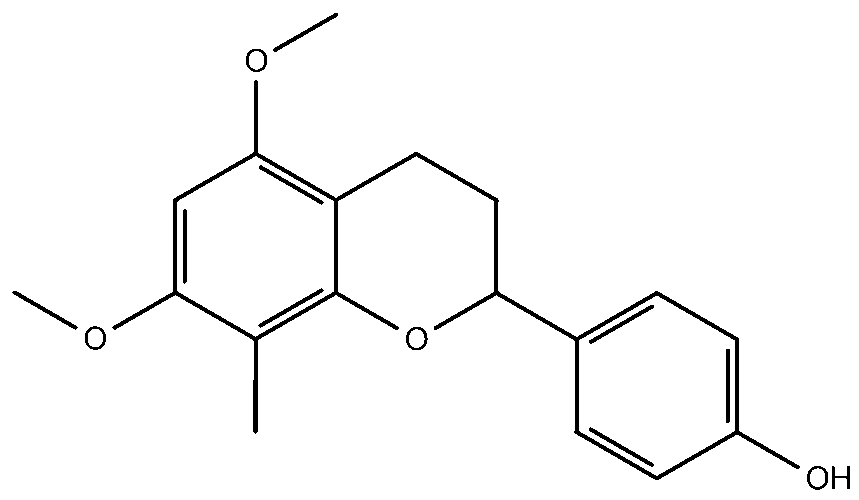 | [11] |
| 33 | Baicalein | 11212 ± 200 μM | 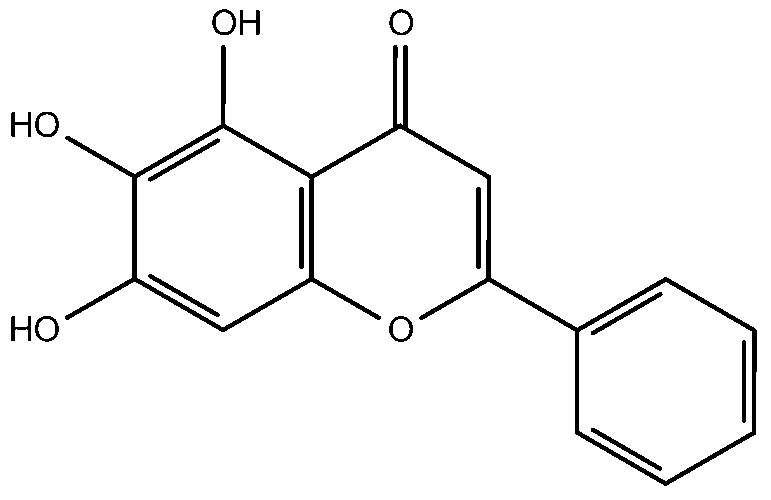 | [12] |
| 34 | Salicylic acid | 13032 μM | 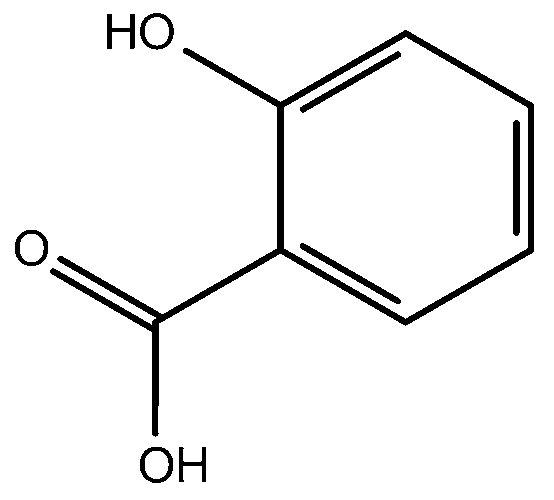 | [13] |
| 35 | Chrysin | 15301 ± 374 μM | 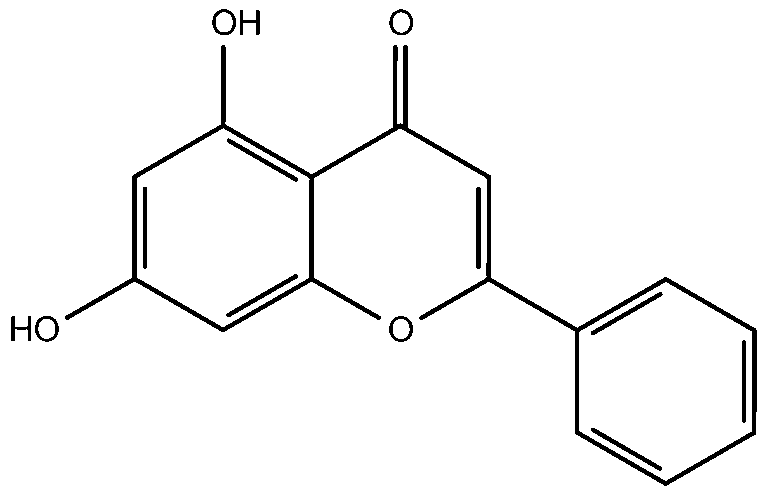 | [12] |
| 36 | Caffeic acid | 19427 μM | 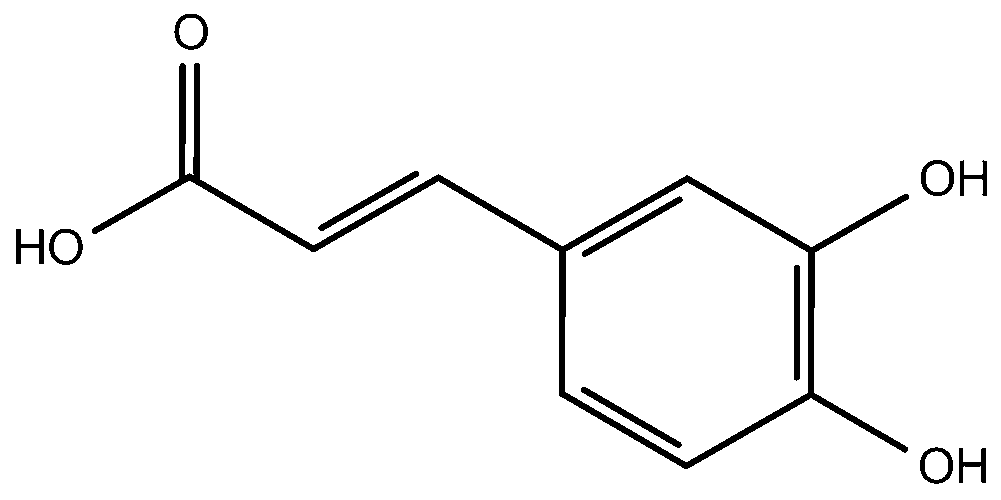 | [13] |
| 37 | Isorhamnetin | 24948 ± 427 μM | 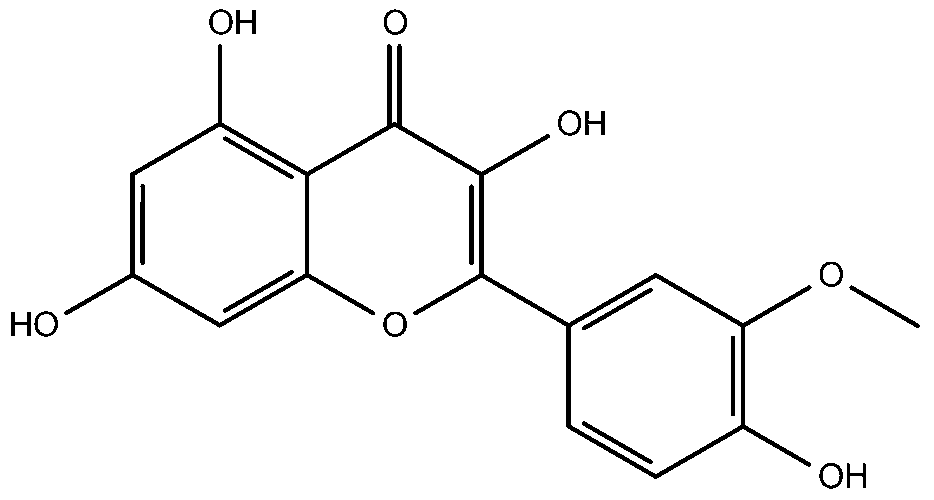 | [12] |
| 38 | p-Coumaric acid | 34113 μM | 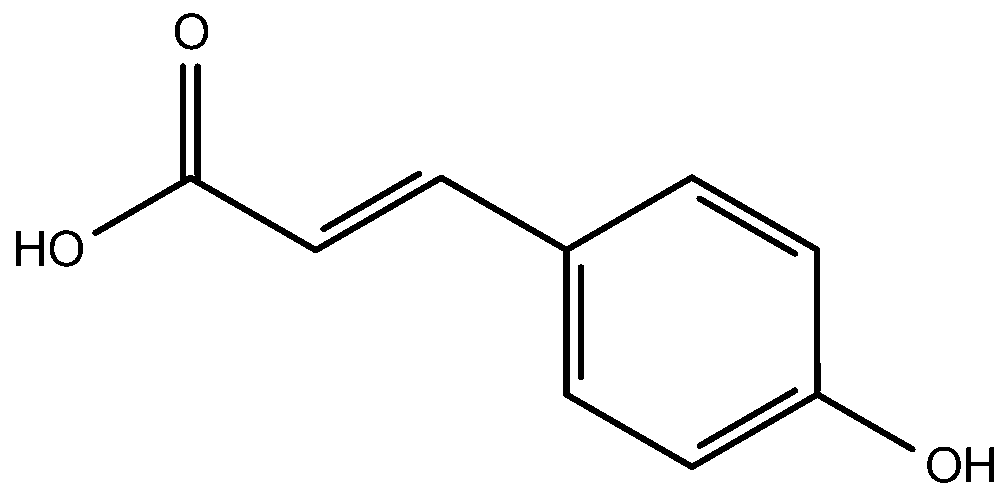 | [13] |
| 39 | Sinapic acid | 37019 μM | 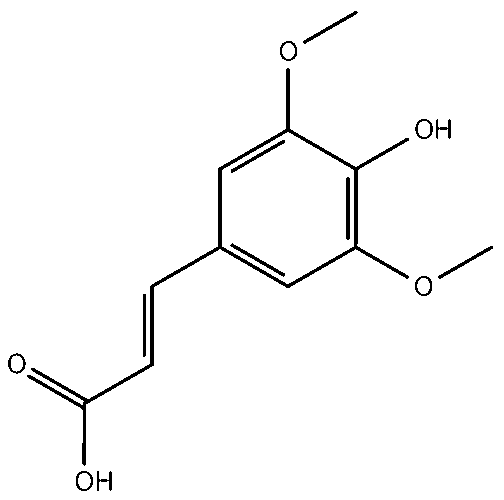 | [13] |
| 40 | Vanillin | 70983 μM | 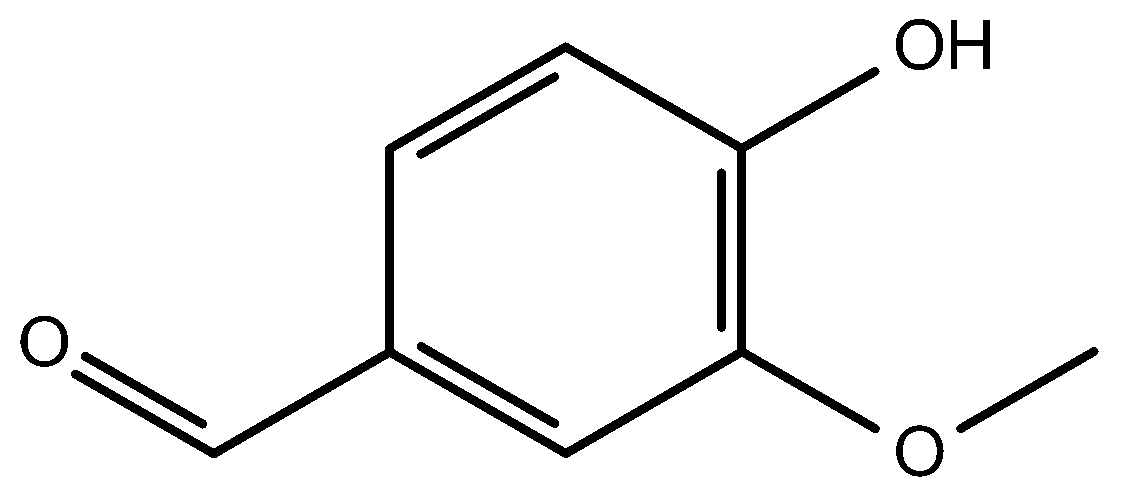 | [13] |

**Table S2** Fragmentation pattern of the major four identified compounds in derivatized Egyptian propolis sample.

| Peak  No. # | Compound Name | Characteristic Fragments m/z (Relative Abundance) |
| --- | --- | --- |
| 4 | D-fructose | 73 (96.8%), 147 (82%), 191 (21.7%), 204 (100%), 217 (91.5%), 437 (95.9%) |
| 8 | D-glucopyranose | 73 (94.4%), 169 (5.6%), 191 (62%), 204 (100%), 217 (83%), 307 (19.3%), 331 (2.1%), 361 (3.2%), 435 (5.8%) |
| 12 | Hexopyranose | 73 (62.5%), 191 (45.4%), 204 (100%), 217 (55%) |
| 27 | Chrysin | 311 (52%), 327 (6.9%), 383 (100%), 399 (3.9%), 471 (3.6%) |

**Table S3** Top twenty compounds resulting from virtual screening of the in-house database of Egyptian propolis phytoligands with human pancreatic α-amylase (PDB ID: 4GQR).

| **Rank** | **Hit name and chemical structure** | **Docking Score** | **Interaction Type** | **Amino acid (ligand functional group) participating in interaction** |
| --- | --- | --- | --- | --- |
| **-** | **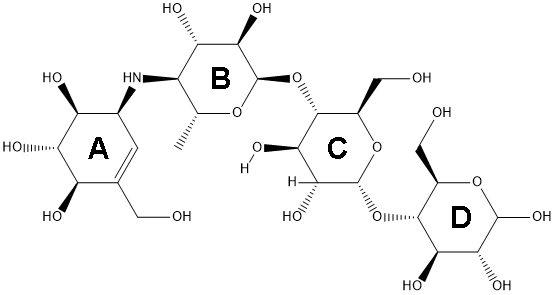**  **Acarbose (Reference Drug)** | -7.5 | H-bond  Hydrophobic | GLN63 (2-OH Ring B), THR163 (3-OH Ring B), ASP197 (6-OH Ring D), GLU233 (2-OH Ring D), HIS299 (3-OH Ring D), ASP300 (6-OH Ring C)  ILE51 (Ring A), ASN53 (Ring A), PRO54 (Ring A), TRP58 (Ring C), TRP59 (Ring B), TYR62 (Ring C), VAL98 (Ring B), HIS101 (Ring D), GLY104 (Ring B), ALA106 (Ring A), VAL107 (Ring A), LEU162 (Ring D), ARG195 (Ring D), ALA198 (Ring D), PHE256 (Ring D), HIS305 (Ring C) |
| **1** | **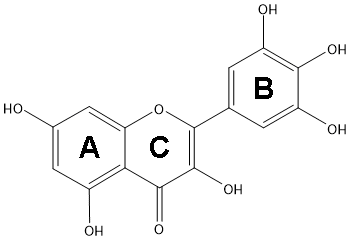**  **Myricetin (Cocrystallized Ligand)** | -10.204 | H-bond  π – π stacking  Hydrophobic  Polar  Charged negative  Charged positive | TRP59 (7-OH), GLN 63 (7-OH), THR 163 (5-OH), ASP 197 (3’-OH), HIE299 (3’-OH), GLU233 (4’-OH), ASP300 (5’-OH)  TRP59 (Ring A)  TRP58 (Ring C), TRP59 (Ring A), TYR62 (Ring A), LEU165 (Ring A), PHE256 (Ring B), ILE235 (Ring B)  GLN63 (Ring B), THR163 (Ring B)  GLU60 (Ring A), ASP197 (Ring B), GLU233 (Ring B), ASP300 (Ring B)  ARG195 (Ring B) |
| **2** | **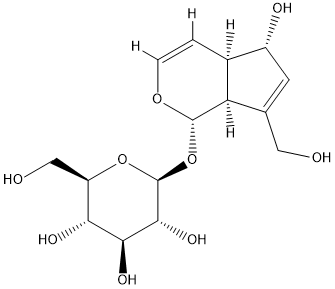**  **Aucubin** | -10.001 | H-bond  Charged negative  Hydrophobic  Polar | TRP59 (6-OH), GLN63 (6-OH), ASP197(6’-OH), HIE299 (6’-OH), GLU233(3’-OH, 4’-OH), ASP300 (2’-OH)  GLU60 (Iridoid nucleus), ASP197 (Sugar), GLU233 (Sugar), ASP300 (Sugar)  TRP59 (Iridoid nucleus), TYR62 (Iridoid nucleus), LEU162 (Iridoid nucleus)  GLN63 (Iridoid nucleus), HIE101 (Iridoid nucleus), THR163 (Iridoid nucleus), ASN298 (Sugar), HIE299 (Sugar) |
| **3** | **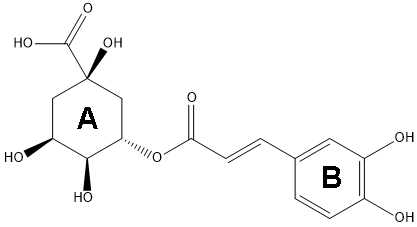**  **Chlorogenic acid** | -9.421 | H-bond  Charged negative  Charged positive  Hydrophobic  Polar | GLN63 (COO-), THR163 (1-OH), GLU233 (3’-OH,  4’-OH)  ASP197 (Caffeic acid moiety), GLU233 (Caffeic acid moiety), ASP300 (Caffeic acid moiety)  ARG195 (Caffeic acid moiety)  TRP58 (Caffeic acid moiety), LEU162 (Quinic acid moiety), LEU165 (Quinic acid moiety), ALA198 (Caffeic acid moiety)  GLN63 (Quinic acid moiety), THR163 (Quinic acid moiety), HIE101 (Caffeic acid moiety), HIE299 (Caffeic acid moiety) |
| **4** | **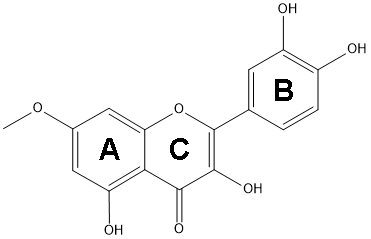**  **Quercetin-7-methyl ether** | -8.535 | H-bond  π – π stacking  Hydrophobic  Polar | GLN63(C=O), ASP197 (4’-OH)  TRP59 (Ring A)  TRP58 (Ring C), TRP59 (Ring C), ALA198 (Ring B)  GLN63 (Ring C) |
| **5** | **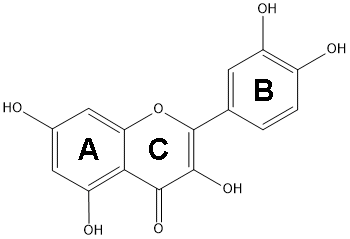**  **Quercetin** | -8.262 | H-bond  π – π stacking  Hydrophobic  Polar | GLN63(C=O), ASP197 (4’-OH)  TRP59 (Ring A)  TRP58 (Ring C), TRP59 (Ring C), ALA198 (Ring B)  GLN63 (Ring C) |
| **6** | **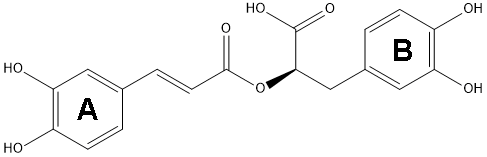**  **Rosmarinic acid** | -8.215 | H-bond  Charged negative  Hydrophobic | ASP197(3-OH,4-OH), ASP300 (3’-OH, 4’-OH), GLN63(9 C=O)  ASP300 (Ring B)  TRP58 (Ring A), TRP59 (Ring A), ALA198 (Ring A) |
| **7** | **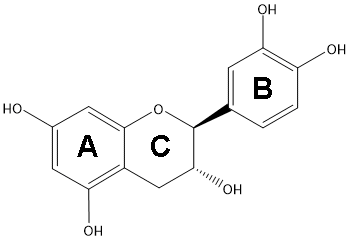**  **Catechin** | -8.109 | H-bond  π – π stacking  Hydrophobic | TYR62 (3-OH), GLU233 (3’-OH)  TRP59 (Ring A)  TRP58 (Ring A), TRP59 (Ring A), TYR62 (Ring C), ALA198 (Ring B) |
| **8** | **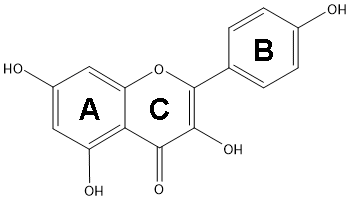**  **Kaempferol** | -8.1 | H-bond  Hydrophobic | GLN63 (C=O) and ASP300 (4’-OH)  TRP58, TRP59, TYR62, HIS101, LEU162, LEU165, ARG195, ALA198, GLU233, ILE235, HIS299 |
| **9** | **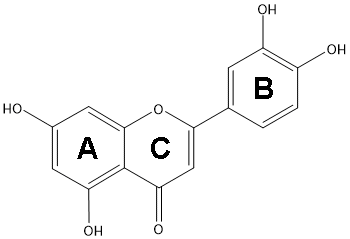**  **Luteolin** | -7.74 | H-bond  π – π stacking  Hydrophobic | ASP197 (3’-OH), HIE299 (3’-OH), GLU233 (4-OH)  TRP58 (Ring B), TYR62 (Ring B), TRP59 (Ring A)  TRP58 (Ring C), TRP59 (Ring C), LEU165 (Ring C), TYR62 (Ring B), |
| **10** | **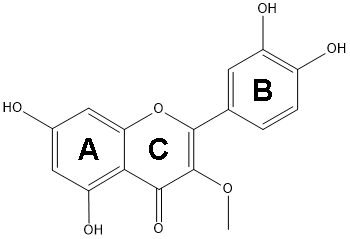**  **Quercetin-3-methyl ether** | -7.65 | H-bond  π – π stacking  Hydrophobic | ASP197 (3’-OH), HIE299 (3’-OH), GLU233 (4’-OH)  TRP58 (Ring B), TYR62 (Ring B), TRP59 (Ring A)  TYR62 (Ring B), LEU162 (Ring C), LEU165 (Ring C)  TYR62 (Ring B), LEU162 (Ring C), LEU165 (Ring C), ILE235 (Ring B) |
| **11** | **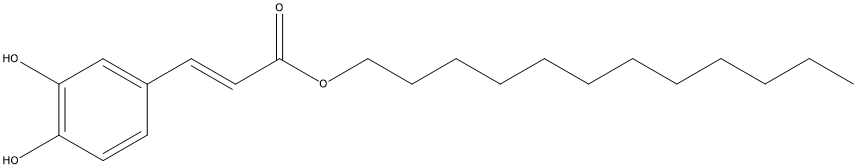**  **Dodecyl caffeate** | -7.298 | H-bond  Hydrophobic | GLN63 (C=O), GLU233 (3-OH), GLU233 (4-OH)  TYR58, TRP59, TRP62 (side chain) |
| **12** | **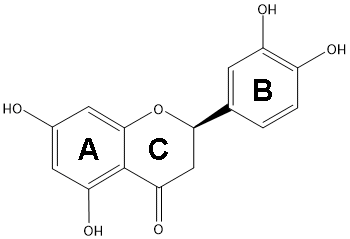**  **Eriodictyol** | -7.293 | H-bond  π – π stacking | GLN63 (5-OH), ARG195 (3’-OH), ASP197 (3’-OH), HIE299 (3’-OH), GLU233 (4’-OH)  TRP59 (Ring A), TYR62 (Ring B) |
| **13** | **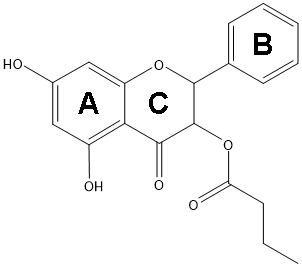**  **5,7-Dihydroxy-3-butanoyloxyflavanone** | -7.279 | H-bond  π – π stacking  Hydrophobic | GLN63 (butanoyl C=O), HIP305 (7-OH)  TRP59 (Ring A)  TYR 62, VAL107, LEU165 (butanoyl side chain) |
| **14** | **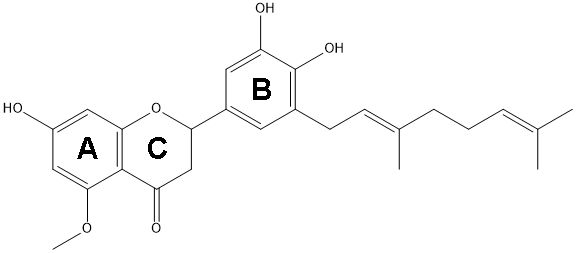**  **isonymphaeol-D** | -7.262 | H-bond  Hydrophobic | THR163 (3’-OH), ASP197 (7-OH)  LEU162, ALA198, VAL98 (Ring A) |
| **15** | **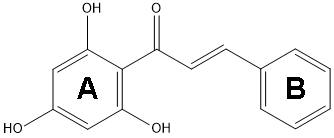**  **Pinocembrin chalcone** | -7.23 | H-bond  π – π stacking | TRP59 (2’-OH), GLN63 (2’-OH), THR163 (6’-OH)  TRP59 (Ring B) |
| **16** | **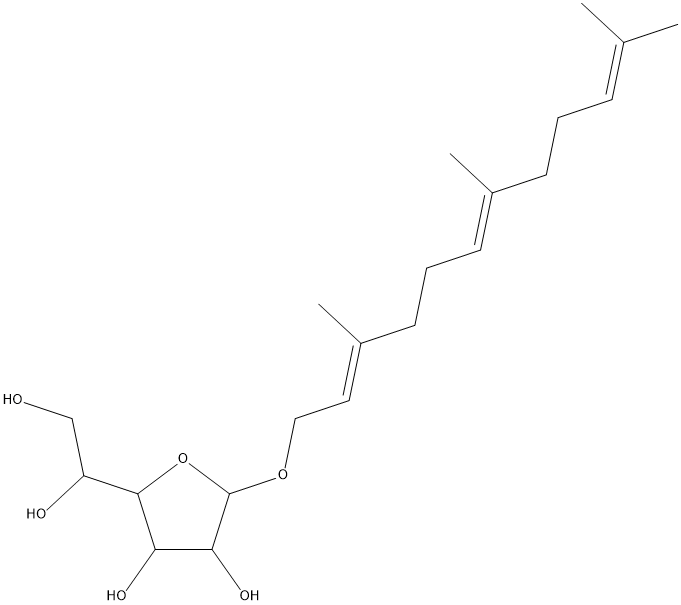**  **beta-D-Mannofuranoside, farnesyl** | -7.181 | H-bond | ASP197 (3-OH), GLU233 (4-OH), GLU233 (7-OH) |
| **17** | **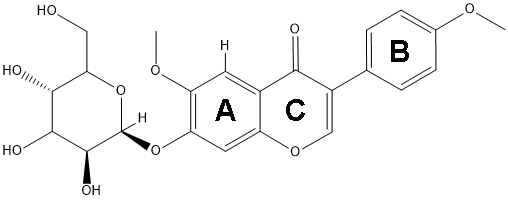**  **4’,6-Dimethoxyisoflavone-7-O-Beta-D-glucopyranoside** | -7.124 | H-bond  π – π stacking | ASP197 (2’’-OH), GLU233 (4’’-OH), HIE299 (6’’-OH)  TRP59 (Ring B) |
| **18** | **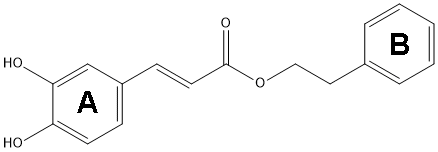**  **Phenylethyl-trans-caffeate** | -7.122 | H-bond  π – π stacking  Hydrophobic  π – cation | GLN63 (C=O), ASP197 (6-OH)  HIP305 (Ring B)  VAL98, ALA198 (Ring A)  HIP305 (Ring B) |
| **19** | **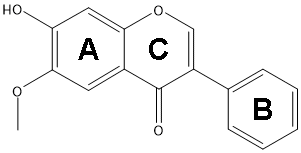**  **7-Hydroxy-6-methoxyisoflavone** | -7.097 | H-bond  π – π stacking  Hydrophobic | TRP59 (7-OH), GLN63 (7-OH)  HIS201 (Ring B)  TRP59, TYR62, LEU165 (Ring A) |
| **20** | **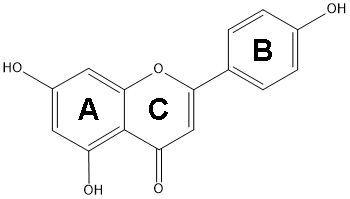**  **Apigenin** | -7.086 | H-bond  π – π stacking  hydrophobic | TRP59(4’-OH), GLN63 (4’-OH)  HIS201 (Ring A)  ALA198 (Ring A), ILE235 (Ring A) |

**References:**

1. Mogoşanu G, Grumezescu A, Mihaiescu D, Istrati D, Mogosanu D, Buteicǎ S. Identification of sugars from Silene Albae Herba using GC-MS technique. UPB Sci Bull Ser B Chem Mater Sci. 2011;73:101–8.

2. Kim SJ, Kumar AP, Lee YI. Enhanced detection and structural characterization of flavonoids by complexation with N,O-bis(trimethysilyl)trifluoroacetamide using electrospray ionization mass spectrometry. Anal Sci. 2008;24:1177–82.

3. Tian J-L, Si X, Wang Y-H, Gong E-S, Xie X, Zhang Y, et al. Bioactive flavonoids from Rubus corchorifolius inhibit α-glucosidase and α-amylase to improve postprandial hyperglycemia. Food Chem. 2021;341:128149.

4. Gao J, Xu P, Wang Y, Wang Y, Hochstetter D. Combined Effects of Green Tea Extracts, Green Tea Polyphenols or Epigallocatechin Gallate with Acarbose on Inhibition against α-Amylase and α-Glucosidase in Vitro. Molecules. 2013;18:11614–23.

5. Sui X, Zhang Y, Zhou W. In vitro and in silico studies of the inhibition activity of anthocyanins against porcine pancreatic α-amylase. J Funct Foods. 2016;21:50–7.

6. Akkarachiyasit S, Yibchok-Anun S, Wacharasindhu S, Adisakwattana S. In Vitro Inhibitory Effects of Cyandin-3-rutinoside on Pancreatic α-Amylase and Its Combined Effect with Acarbose. Molecules. 2011;16:2075–83.

7. Tiji S, Bouhrim M, Addi M, Drouet S, Lorenzo JM, Hano C, et al. Linking the Phytochemicals and the α-Glucosidase and α-Amylase Enzyme Inhibitory Effects of Nigella sativa Seed Extracts. Foods. 2021;10.

8. Proença C, Freitas M, Ribeiro D, Tomé SM, Oliveira EFT, Viegas MF, et al. Evaluation of a flavonoids library for inhibition of pancreatic α-amylase towards a structure–activity relationship. J Enzyme Inhib Med Chem. 2019;34:577–88.

9. Liu S, Yu J, Guo S, Fang H, Chang X. Inhibition of pancreatic α-amylase by Lonicera caerulea berry polyphenols in vitro and their potential as hyperglycemic agents. LWT. 2020;126:109288.

10. Aleixandre A, Gil JV, Sineiro J, Rosell CM. Understanding phenolic acids inhibition of α-amylase and α-glucosidase and influence of reaction conditions. Food Chem. 2022;372:131231.

11. Yi J, Zhao T, Zhang Y, Tan Y, Han X, Tang Y, et al. Isolated compounds from Dracaena angustifolia Roxb and acarbose synergistically/additively inhibit α-glucosidase and α-amylase: an in vitro study. BMC Complement Med Ther. 2022;22:177.

12. Zhao Y, Wang M, Huang G. Structure-activity relationship and interaction mechanism of nine structurally similar flavonoids and α-amylase. J Funct Foods. 2021;86:104739.

13. Pollini L, Riccio A, Juan C, Tringaniello C, Ianni F, Blasi F, et al. Phenolic Acids from Lycium barbarum Leaves: In Vitro and In Silico Studies of the Inhibitory Activity against Porcine Pancreatic α-Amylase. Processes. 2020;8.
