# Supplementary material for: Synergistic effect of potential alpha-amylase inhibitors from Egyptian propolis with acarbose using in silico and in vitro combination analysis
Source: BMC Complement Med Ther. 2024 Jan 30;24:65. doi: 10.1186/s12906-024-04348-x (PMC10826043; doi:10.1186/s12906-024-04348-x)
Supplement: Supplementary file 2 — Additional file 2. [file 12906_2024_4348_MOESM2_ESM.pptx]

## Slide 1
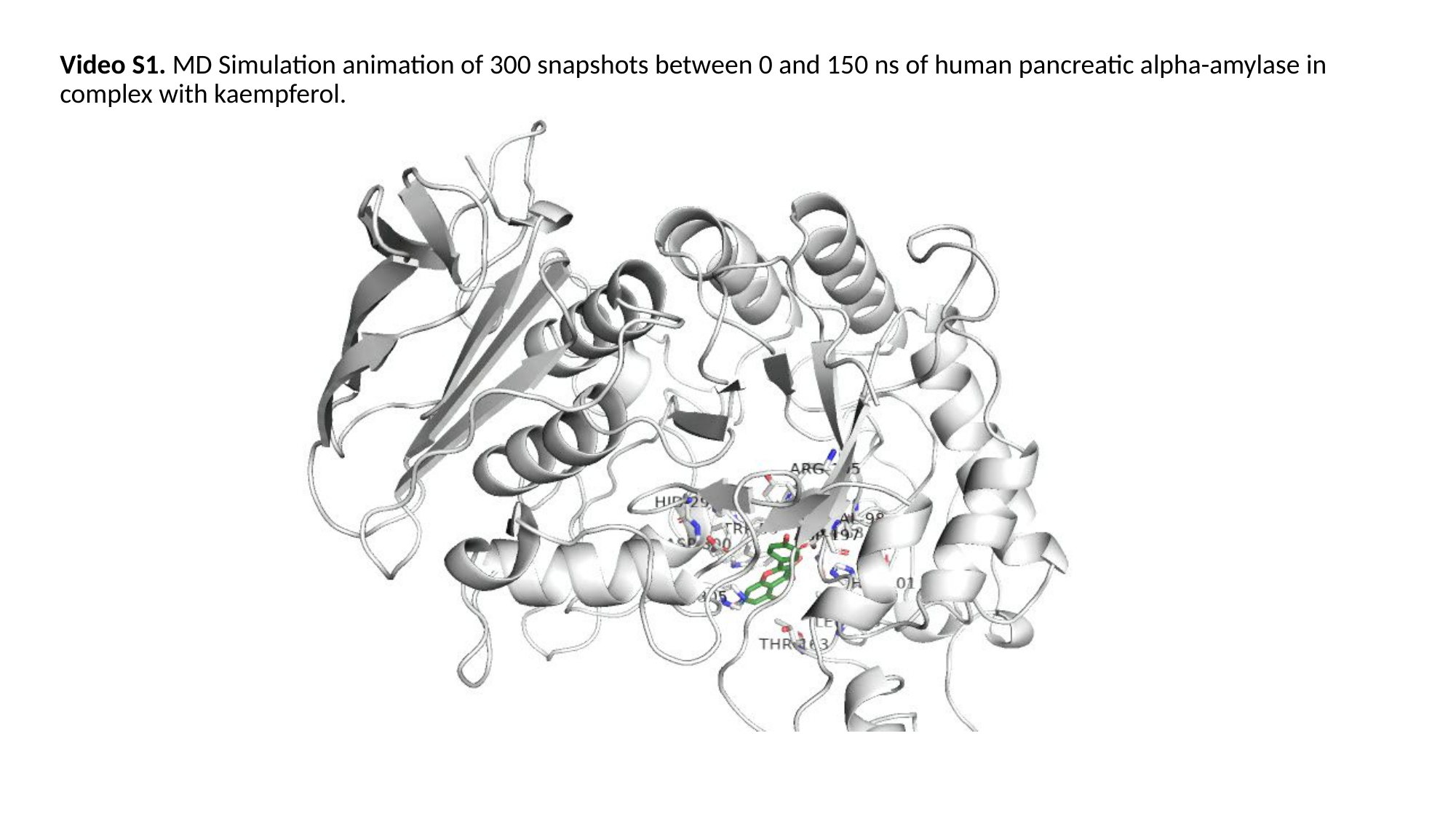

# Video S1. MD Simulation animation of 300 snapshots between 0 and 150 ns of human pancreatic alpha-amylase in complex with kaempferol.

## Slide 2
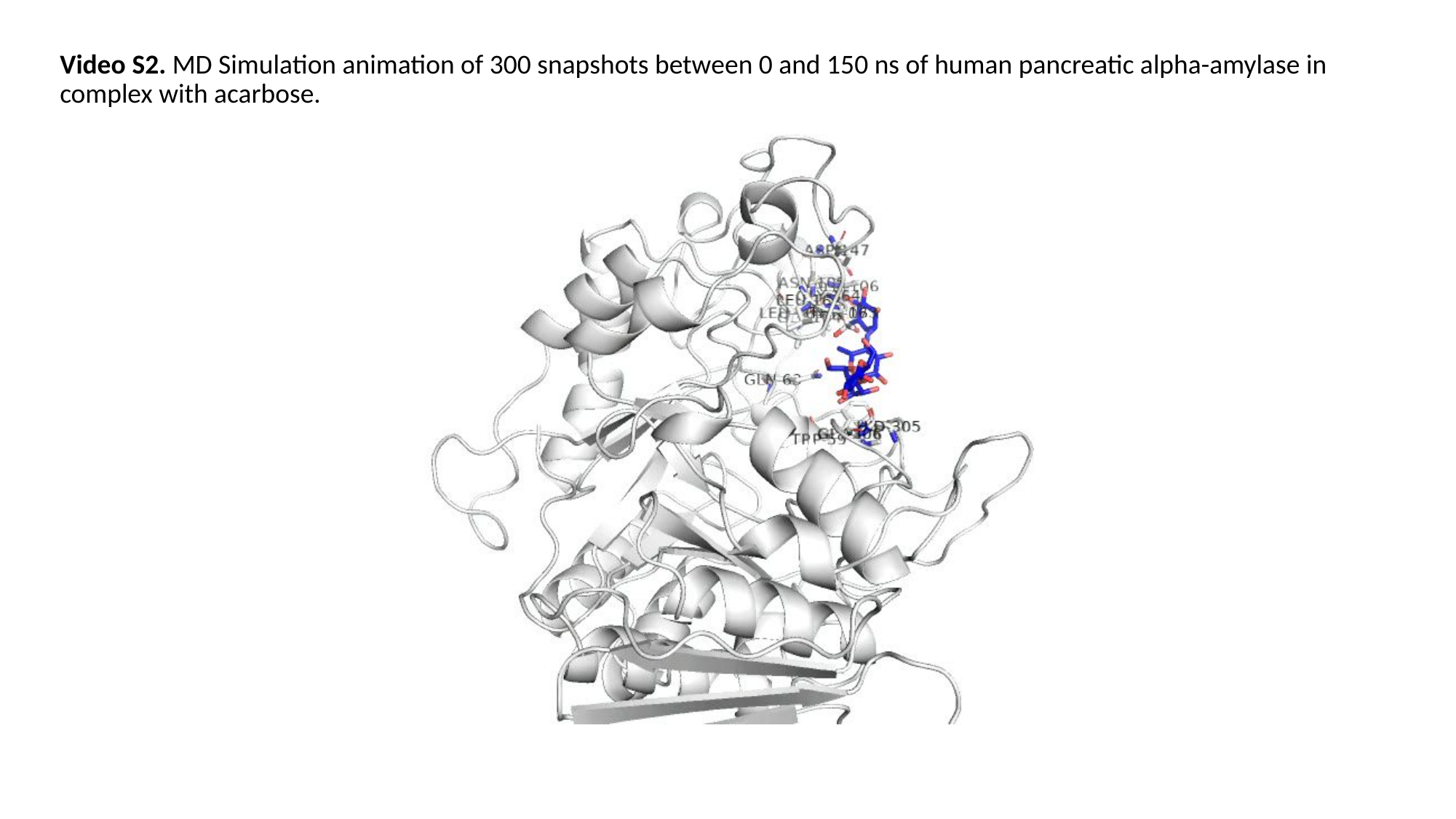

# Video S2. MD Simulation animation of 300 snapshots between 0 and 150 ns of human pancreatic alpha-amylase in complex with acarbose.

## Slide 3
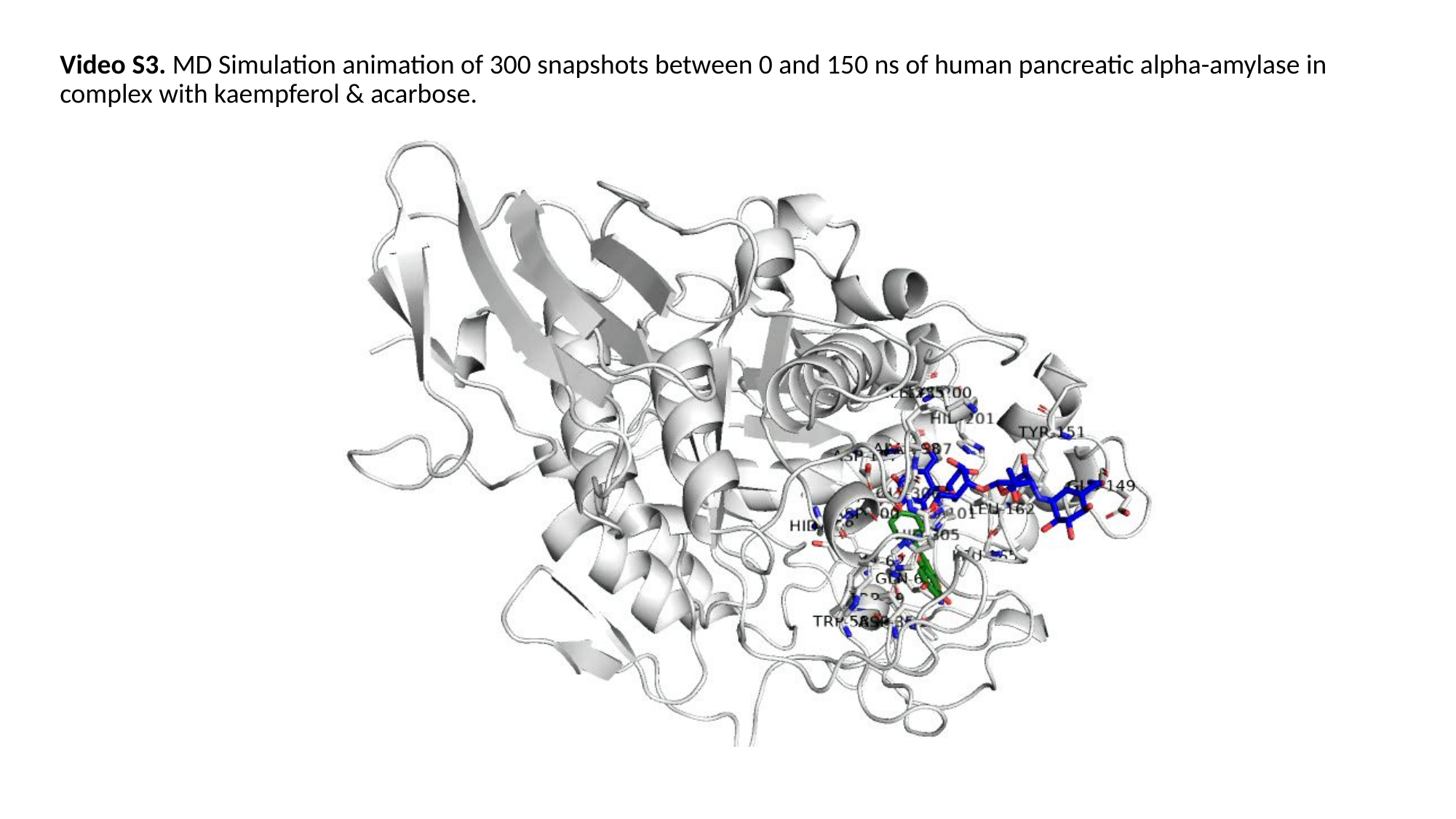

# Video S3. MD Simulation animation of 300 snapshots between 0 and 150 ns of human pancreatic alpha-amylase in complex with kaempferol & acarbose.
